# Comparative structural and evolutionary analyses predict functional sites in the artemisinin resistance malaria protein K13

**DOI:** 10.1038/s41598-019-47034-6

**Published:** 2019-07-23

**Authors:** Romain Coppée, Daniel C. Jeffares, Maria A. Miteva, Audrey Sabbagh, Jérôme Clain

**Affiliations:** 1 Université de Paris, UMR 261 MERIT, IRD, F-75006 Paris, France; 2 Department of Biology and York Biomedical Research Institute, University of York, Wentworth Way, York, YO10 5DD UK; 3 Université de Paris, Inserm U1268 MCTR, CiTCom UMR 8038 CNRS, Paris, France; 4 Centre National de Référence du Paludisme, Hôpital Bichat-Claude Bernard, Assistance Publique des Hôpitaux de Paris, F-75018 Paris, France

**Keywords:** Phylogeny, Protein analysis, Protein function predictions, Molecular evolution

## Abstract

Numerous mutations in the *Plasmodium falciparum* Kelch13 (K13) protein confer resistance to artemisinin derivatives, the current front-line antimalarial drugs. K13 is an essential protein that contains BTB and Kelch-repeat propeller (KREP) domains usually found in E3 ubiquitin ligase complexes that target substrate protein(s) for ubiquitin-dependent degradation. K13 is thought to bind substrate proteins, but its functional/interaction sites and the structural alterations associated with artemisinin resistance mutations remain unknown. Here, we screened for the most evolutionarily conserved sites in the protein structure of K13 as indicators of structural and/or functional constraints. We inferred structure-dependent substitution rates at each amino acid site of the highly conserved K13 protein during the evolution of *Apicomplexa* parasites. We found two solvent-exposed patches of extraordinarily conserved sites likely involved in protein-protein interactions, one in BTB and the other one in KREP. The conserved patch in K13 KREP overlaps with a shallow pocket that displays a differential electrostatic surface potential, relative to neighboring sites, and that is rich in serine and arginine residues. Comparative structural and evolutionary analyses revealed that these properties were also found in the functionally-validated shallow pocket of other KREPs including that of the cancer-related KEAP1 protein. Finally, molecular dynamics simulations carried out on PfK13 R539T and C580Y artemisinin resistance mutant structures revealed some local structural destabilization of KREP but not in its shallow pocket. These findings open new avenues of research on one of the most enigmatic malaria proteins with the utmost clinical importance.

## Introduction

Current efforts to control malaria are threatened by the spread in Southeast Asia (SEA) of *Plasmodium falciparum* parasites that are resistant to artemisinin derivatives (ARTs)^1^. Treatment failures are now reported in some geographic areas of SEA for the current front-line ART-based combination therapies^1–3^. Artemisinin resistance (ART-R) is defined as parasites exhibiting *in vivo* a delayed clearance time following an ART-based treatment^1^ and *in vitro* an increased survival rate following a brief exposure to a high dose of ART^4^. This phenotype is primarily conferred by single non-synonymous mutations in the *P. falciparum k13* (*pfk13*, also named *pfkelch13*) gene^5,6^. Multiple *pfk13* ART-R alleles have emerged concomitantly in the early 2000’s^7^, until a specific, multidrug-resistant lineage carrying the *pfk13* C580Y allele spread and nearly reached fixation, especially in the East Thailand-Cambodia-Lao PDR-Vietnam region^5,7–10^.

**Figure 1:**
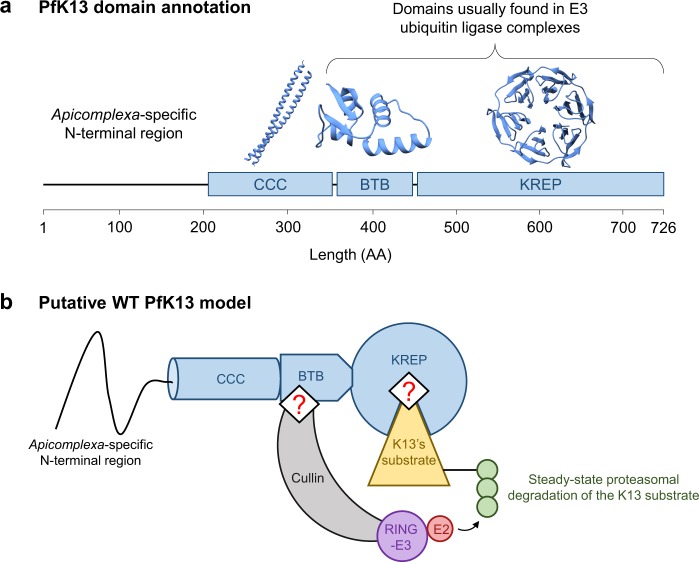
Schematic and structural representation of PfK13 and its putative function as substrate adaptor. (**a**) PfK13 domain annotation. Three domains of PfK13 are annotated in databases: coiled-coil-containing (CCC), BTB, and Kelch-repeat propeller (KREP). The *Apicomplexa*-specific N-terminal domain is predicted to exhibit a random-coil conformation. The crystal structure of BTB and KREP domains was solved; the CCC domain is expected to form two helices coiling together. (**b**) Proposed, simplified model of the protein complex containing PfK13, based on PfK13 domain annotation and co-immunoprecipitation experiments^17,21,22,89,90^. The BTB domain of PfK13 is expected to bind a scaffold Cullin protein, while the KREP domain likely binds to the substrate molecule(s) further ubiquitinated and possibly degraded by the proteasome. Importantly, the regions and sites of PfK13 involved in binding are unknown (represented as red ‘?’ symbol in the figure). For ease of representation, PfK13 was shown as a monomer although the crystal structure of PfK13 BTB-KREP was solved as a dimer.

*pfk13* encodes a 726-amino acid protein (PfK13) consisting of a poorly conserved *Apicomplexa*-specific N-terminal region and three annotated, highly conserved domains^9^ (Fig. 1a): a coiled-coil-containing (CCC; amino acids 212–341), a BTB (Broad-complex, tramtrack and bric-à-brac; also known as BTB/POZ; amino acids 350–437) and a C-terminal Kelch-repeat propeller (KREP; amino acids 443–726) which harbors nearly all *pfk13* alleles associated with ART-R^5,7^. Using large scale mutagenesis screens, the *k13* gene was reported as essential for the intraerythrocytic growth of both *P. falciparum* and *P. berghei* asexual parasites^11,12^. Removal of PfK13, through conditional mislocalization of the protein or diCre-based gene deletion, leads to an arrest at the early intraerythrocytic ring stage followed by a slow transition to condensed parasite forms^13^. The timing of the growth arrest in PfK13-inactivated parasites is consistent with the ART-R phenotype being expressed at the early ring stage^14^. In addition, parasites carrying *pfk13* ART-R mutations exhibit an altered intraerythrocytic cell cycle, with a lengthened ring stage and a shortened trophozoite stage, relative to wild type (WT)^15^. At the molecular level, naturally-occurring *pfk13* mutants associate with several key features: an increased expression of unfolded protein response pathways, detected by the *in vivo* transcriptomics analysis of 1,043 *P. falciparum* isolates from patients with acute malaria response^16^; lower levels of ubiquitinated proteins^17,18^; 2-fold lower abundance of PfK13 protein, as measured by quantitative dimethyl-based proteomics analysis of Cambodian isogenic strains^19^ (note that a different result was reported for two African isogenic strains^20^); and a basal, constitutive phosphorylation of the parasite eukaryotic initiation factor-2α (eIF2α) at the early intraerythrocytic stage, which controls the repression of general translation and ART-induced latency^15,21^. Altogether, it seems that the parasite acquires ART-R by altering its cell cycle such as it spends more time at a developmental stage that is less sensitive to ART drugs (early ring), while at the same time mounting a response mitigating ART-induced damages. Some indications of the interactors with PfK13 partially clarify its function. For example, PfK13 was immunoprecipitated with the phosphatidylinositol 3-kinase (PfPI3K), an enzyme producing the lipid phosphatidylinositol 3-phosphate (PI3P) involved in protein export from the *P. falciparum* endoplasmic reticulum and which cellular levels associate with ART-R^17^. Ubiquitination of PfPI3K and its subsequent proteasomal degradation are decreased in *pfk13* mutant parasites. The increased abundance of PfPI3K, and its lipid product PfPI3P, in mutant parasites may influence host cell remodeling and neutralize ART toxic proteopathy^17,20^.

These experimental data and the Gene Ontology annotation of PfK13, which contains BTB and KREP domains, are consistent with the hypothesis that PfK13 may function as an E3 ubiquitin ligase substrate adaptor (Fig. 1b)^17,21–23^. Many BTB- and/or KREP-containing proteins are found in multi-subunit RING-type E3 ligase complexes in which substrate protein(s) will be ubiquitinated^24–27^. In those complexes, the BTB domain mediates both varying oligomerization architectures and the recruitment of a scaffold Cullin protein which interacts with RING E3 protein, whereas the KREP domain serves as a receptor for substrate protein(s) to be ubiquitinated. The E3 ligase activity of RING then catalyzes the transfer of ubiquitin from the ubiquitin-conjugating enzyme (E2) to the protein substrate(s)^24,28^. Some KREP domains contain at their bottom side a shallow pocket involved in the binding of substrate protein(s)^24^. For example, the shallow pocket of KEAP1 and KLHL3, from the large BTB-Kelch family of proteins, directly binds to the transcription factor Nrf2 and some WNK kinases, respectively, and controls their ubiquitination^29,30^. Importantly, PfK13 is reported to be the sole BTB-KREP-containing protein in *Plasmodium* while mammals include ~60 members, each targeting specific substrate(s)^31^. Therefore, PfK13 may bind several substrates, in addition to PfPI3K.

Although it is expected that PfK13 may act similarly to other BTB-Kelch proteins involved in ubiquitination activities^17^, the putative binding regions and functionally important sites of PfK13 and the structural alteration(s) associated with PfK13 ART-R mutations remain uncharacterized (Fig. 1b). Because of its high conservation^9^ and essentiality across *Apicomplexa* species^11–13,32^, the function of K13 likely remained unchanged during *Apicomplexa* evolution. As a consequence, we would expect that amino acid replacements at the functional sites of K13 would be strongly detrimental to protein function, and would be purged by purifying selection. To find the most conserved amino acid sites of K13, we inferred substitution rates for each amino acid site of the WT K13 sequence from 43 *Apicomplexa* species, taking into account both the spatial correlation of site-specific substitution rates in the protein tertiary structure and the species phylogeny^33^. We identified in BTB and KREP two solvent-exposed patches of extraordinarily conserved sites, likely involved in protein-protein interactions. As controls, we found similarly evolutionarily conserved and located patches in several BTB- and KREP-containing proteins found in mammals, including KEAP1. Finally, we tested through molecular dynamics simulations whether the R539T and C580Y ART-R mutations in PfK13 altered some structural properties of the KREP domain and its putative binding sites.

## Results

### K13 sequence sampling, multiple alignment and phylogenetic reconstruction

Forty-three amino acid sequences from distinct *Apicomplexa* species were unambiguously identified as orthologous to PfK13 in sequence databases, encompassing 21 *Plasmodium* and 22 other *Apicomplexa* K13 sequences (*Cryptosporidia*, n = 7; *Piroplasmida*, n = 7; and *Coccidia*, n = 8). The length of K13 protein sequences ranged from 506 (*Eimeria brunetti*) to 820 (*Hammondia hammondi*) amino acids (Supplementary Table S1). By visual inspection, the three annotated domains of K13 (CCC, BTB and KREP) were very conserved (see the K13 sequence alignment in Supplementary Fig. S1), whereas the N-terminal region preceding the CCC domain appeared much more variable in length and amino acid composition, to a point of not being even detected in databases for many non-*Plasmodium Apicomplexa* species. Since K13 sequences aligned poorly in that region, the first 234 amino acid positions of the K13 multiple alignment were removed, along with other positions showing a high divergence level and/or gap enrichment among sequences (32 amino acid positions; Supplementary Fig. S1). The final K13 multiple sequence alignment contained 514 amino acid positions which covered the whole CCC, BTB and KREP domains. The average pairwise sequence identity in that cleaned K13 sequence alignment was 64.6%, ranging from 48.6% for the *Babesia bigemina*-*Cryptosporidium ubiquitum* comparison to 99.2% for the *P. chabaudi* spp. pair. This is well below the average pairwise sequence conservation found among *Plasmodium* species (84.9%, with a minimum value of 78.3% for the *P. vivax*-*P. chabaudi adami* pair).

The maximum-likelihood phylogenetic tree built from the curated alignment of the corresponding *Apicomplexa k13* cDNA sequences revealed four monophyletic groups: *Cryptosporidia*, *Plasmodium, Piroplasmida* and *Coccidia*, all supported by high bootstrap values (≥98%; Supplementary Fig. S2). The group of *Hematozoa k13* sequences appeared as paraphyletic (bootstrap value = 100%), with *Piroplasmida* unexpectedly clustering with *Coccidia* (Supplementary Fig. S2). The phylogenetic relationships of *Plasmodium k13* sequences were largely consistent with the acknowledged phylogeny of *Plasmodium* species, except for bird-infecting *Plasmodium k13* sequences (*P. gallinaceum* and *P. relictum*), which appeared related to human-infecting *P. ovale* spp. sequences, although this grouping was poorly supported (bootstrap value = 47%; Supplementary Fig. S2).

### The *k13* sequence has evolved under strong purifying selection

To evaluate the selective pressure acting on *k13* which reflects its level of conservation during evolution, we used codon substitution models to estimate the rate of non-synonymous to synonymous substitutions, *ω* = *d*_N_*/d*_S_, across codon sites of the *k13* sequence (site models) and branches of the *k13* phylogeny (branch models). A series of nested likelihood ratio tests (LRTs) using different sets of site and branch models were carried out using the codeml tool from the PAML package^34^. When applied to the *k13* codon alignment, LRTs of codon and branch models indicated varying substitution rates *ω* both across codon sites of the *k13* sequence (M0-M3 comparison, *p* = 3.3 × 10^−225^) and among branches of the *k13* phylogeny (M0-FR, *p* = 1.9 × 10^−53^; Tables 1 and S2). This suggests that *k13* has evolved under a variable selective regime both across codon sites and among lineages. No evidence of positive selection was found in any of the tree branches (Supplementary Fig. S3). Similarly, site models incorporating positive selection (M2a and M8) provided no better fit to the data than those including only purifying selection and neutral evolution (M1a and M7: 2Δℓ = 0 in both cases; Table 1), thus supporting an absence of detectable adaptive selection events at any *k13* codon site over the long time scale of *Apicomplexa* evolution. Altogether, the data indicate that much of the K13 protein, except the N-terminal region which was not studied here, has been strongly conserved over evolutionary time.

**Table 1:** Lack of detectable positive selection in the *k13* gene using codon substitution models.

Models	Purpose of the test	2Δℓ	df	*p value*
M0-FR	Heterogeneity of *ω* among lineages of the phylogenetic tree	457.0	82	1.9 × 10^−53^
M0-M3	Heterogeneity of *ω* across codon sites	1,046.3	4	3.3 × 10^−225^
M1a-M2a	Detection of sites evolving under positive selection	0	2	1
M7-M8	Detection of sites evolving under positive selection	0	2	1

*ω*, non-synonymous (*d*_N_) to synonymous (*d*_S_) substitution rate ratio (*d*_N_/*d*_S_); *2∆*ℓ, log-likelihood ratio of the two tested models; df, degrees of freedom. 2Δℓ was compared to a chi-squared table to determine the significance of the likelihood ratio tests. The M1a-M2a comparison is reported to be more stringent than the M7-M8 comparison^34^.

**Figure 2:**
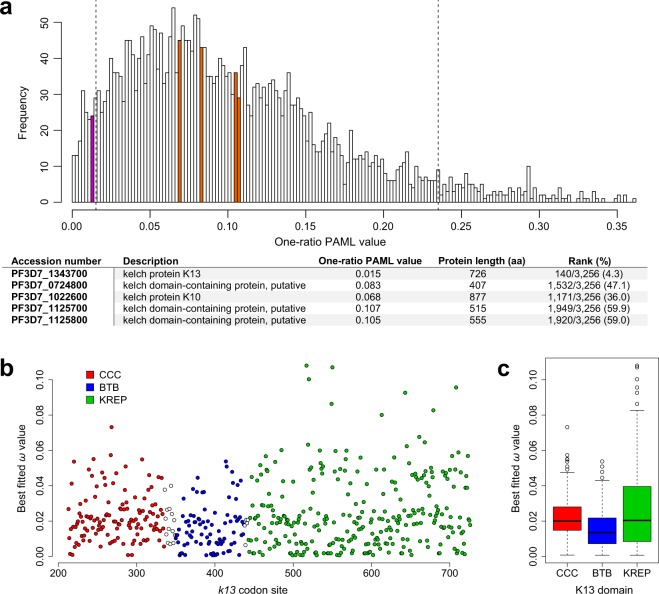
Conservation level of the K13 protein, domains and codon sites. (**a**) Conservation level of *k13* compared to those of all protein-coding genes of *Plasmodium*. The one-ratio PAML values, calculated for 3,256 orthologous genes among six *Plasmodium* species (*P. falciparum*, *P. berghei*, *P. chabaudi*, *P. vivax*, *P. yoelii* and *P. knowlesi*), were retrieved from the study of Jeffares and colleagues^35^. Magenta and orange bars indicate the location of *k13* and four other KREP protein-coding genes (PlasmoDB accession numbers: PF3D7_1022600, PF3D7_1125800, PF3D7_0724800, PF3D7_1125700) respectively. Vertical dashed lines show the 5% cutoff of the most and less conserved protein-coding genes. Among KREP protein-coding genes, only *k13* belonged to the top 5% of the most conserved protein-coding genes of the *Plasmodium* proteome. The table shown below the histogram provides the rank of each KREP-containing protein-coding sequence in the whole dataset. (**b**) Conservation level of the *k13* codon sites. The scatter plot shows the conservation level of *k13* codon sites using the *pfk13* sequence as reference for the codon numbering (starting at codon 213). White circles correspond to inter-domain positions. All codon sites were reported to evolve under strong purifying selection, with *ω* drastically <1. We used the *ω* estimates obtained under the best fitted PAML model M3 that indicates a variable selective regime among codon sites. (**c**) Conservation level of the annotated K13 domains. The box-whisker plot shows that BTB evolves under more intense purifying selection compared to either CCC or KREP, using non-parametric Mann-Whitney *U* test (*p* < 0.05). Box boundaries represent the first and third quartiles and the length of whiskers correspond to 1.5 times the interquartile range.

When considering the one-ratio PAML value for 3,256 protein-coding genes previously estimated by Jeffares *et al*.^35^ using six *Plasmodium* species, *k13* ranked among the 5% most conserved protein-coding genes of the *Plasmodium* proteome (rank: 140/3,256; Fig. 2a). Since a significant correlation between protein length and one-ratio PAML value was evidenced in the whole dataset (Spearman’s rank correlation: *p* = 9.2 × 10^−82^, r = 0.33; Supplementary Fig. S4), we repeated the analysis by considering only those protein sequences whose length was included in an interval of ±100 amino acids centered on the PfK13 protein length (Spearman’s rank correlation: *p* = 0.83, r = 0.01). Again, *k13* ranked among the most conserved protein-coding genes of the *Plasmodium* proteome (sized-rank: 6/393), whereas four other five- or six-bladed KREP protein-coding sequences showed much less intense levels of conservation than *k13* (Figs. 2a and S5).

### Variable levels of amino acid conservation between the annotated domains of K13

We then compared the conservation level between the annotated domains of K13 (CCC, BTB and KREP) using *ω* estimates obtained under the best fitted PAML model M3 that indicates a variable selective regime among codon sites (Supplementary Table S2). First, we noted that the three domains have evolved under strong purifying selection with most codon sites being highly conserved during evolution (Fig. 2b). BTB was however found to evolve under more intense purifying selection than either CCC (*p* = 1.6 × 10^−4^, Mann-Whitney *U* test) or KREP (*p* = 1.0 × 10^−3^), and no difference in *ω* estimates was detected between CCC and KREP (*p* = 0.75; Fig. 2c). To confirm these results, we inferred site-specific substitution rates at the protein level using the FuncPatch server which considers their spatial correlation in protein tertiary structure (hereafter called *λ* substitution rates)^33^. *λ* could not be inferred for the CCC domain because of the lack of a resolved 3D structure. The analysis confirmed that BTB was more conserved than KREP during *Apicomplexa* evolution (*p* = 5.4 × 10^−5^, Mann Whitney *U* test).

### The BTB domain of K13 resembles that of KCTD proteins and exhibits a predicted functional patch

We then performed a more extensive study of the BTB and KREP domains of K13 because of their likely role in mediating K13 functions and the availability of their tertiary structures. To detect patches of slowly evolving amino acid sites in the BTB-KREP structure, we focused on the site-specific substitution rates *λ* at the amino acid level mentioned above. *λ*, estimated using FuncPatch, has been shown to provide a more reliable estimation of the conservation level at amino acid sites compared to standard substitution estimates, especially in the case of highly conserved proteins^33,36^.

**Figure 3:**
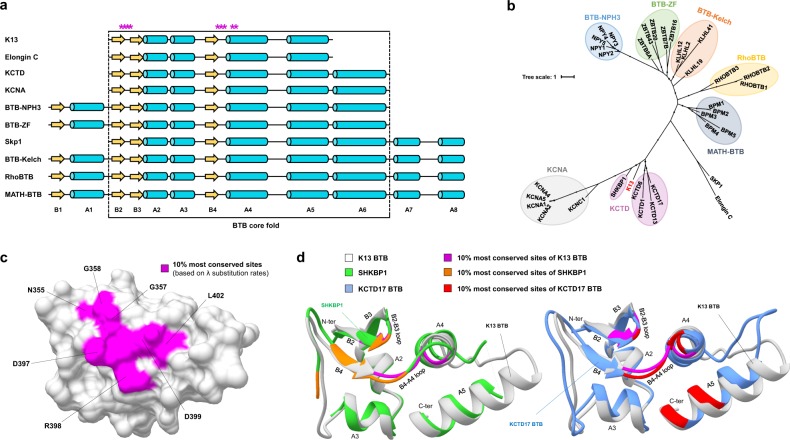
Conservation and structure homology of the K13 BTB fold. (**a**) Linear schematic representation of the BTB fold of some BTB-containing protein families. Yellow arrows and cyan cylinders represent strands and helices, respectively. Stars in magenta correspond to the 10% most conserved K13 BTB amino acid sites (based on the ranking of the *λ* substitution rates, FuncPatch analysis; Supplementary Dataset S1). Structural elements are labelled. (**b**) Maximum-likelihood unrooted phylogenetic tree of the BTB core fold using a few reference sequences per BTB-containing protein family. Each BTB-containing protein family forms a monophyletic group, identified with a colored background. K13 BTB is written in red color and clusters with the KCTD protein family of BTB-containing proteins. (**c**) Patch of slowly evolving amino acid sites in a three-dimensional view of PfK13 BTB. The amino acid sites are labelled using the PfK13 sequence as reference. (**d**) Superposition of the BTB fold of K13 with that of two members of the KCTD protein family. The most conserved amino acid sites for each protein was based on FuncPatch analysis and are shown in magenta, orange and red for K13, SHKBP1 and KCTD17, respectively. Other amino acid sites are shown in white, green, and cornflower blue for K13, SHKBP1 and KCTD17, respectively. Three common positions were identified in the BTB conserved patches of K13, SHKBP1 and KCTD17: 357 on the B2-B3 loop and 397–398 on the B4-A4 loop.

Although K13 shares similarities with the BTB-Kelch family of proteins that are commonly found in E3 ubiquitin ligase complexes^5^, the BTB domain of K13 exhibited atypical features compared to this protein family. First, the K13 BTB fold appeared shortened, lacking the A6 helix and the N- and C-terminal extensions^26^ (Fig. 3a), similarly to the Elongin C protein which acts as an E3 ubiquitin ligase adaptor mediating the ubiquitination of substrates^37^. Second, the primary BTB sequence of K13 grouped with those of the potassium (K+) channel tetramerization domain (KCTD) protein family (Fig. 3b). Finally, the BTB of K13 exhibited a higher similarity in tertiary structure with that of KCTD17 (used as reference for KCTD proteins) compared to those of Elongin C and KEAP1 (the later used as reference for BTB-Kelch proteins): the root-mean-square deviations (RMSDs) of atomic positions for pairs of superimposed BTB domains were 1.13 Ångström (Å) for K13-KCTD17, 2.33 Å for K13-Elongin C and 2.17 Å for K13-KEAP1. In all, these results suggest that K13 BTB is related in sequence and structure to that of KCTD proteins. KCTDs are soluble non-channel proteins containing a BTB domain (but no KREP) involved in variable oligomerization architectures and often acting as versatile scaffold in E3 ubiquitin ligase complexes^27^.

To identify putative functional sites in K13 BTB, we examined whether a spatial correlation of the site-specific substitution rates *λ* was present in the K13 BTB-KREP tertiary structure. Despite a low standard deviation of substitution rates across amino acid sites, a significant spatial correlation was found (Table 2). The 10% most conserved sites predicted by FuncPatch formed one clearly bounded patch on the BTB surface and consisting of sites located at the B2-B3 and B4-A4 loops and at the A4 helix (positions/residues 355–358/NVGG, 397–399/DRD and 402–403/LF using the PfK13 sequence numbering; Fig. 3c). To test whether a similarly located, conserved patch was also found in the BTB domain of KCTD proteins – to which K13 BTB is the most similar – we inferred site-specific substitution rates *λ* from 124 and 139 orthologous sequences of SHKBP1 and KCTD17, acting as E3 ubiquitin ligase adaptors mediating the ubiquitination of substrates^27^. For both proteins, the 10% most conserved BTB positions formed a patch that partially overlapped with the one of K13, with positions 357 (B2-B3 loop), 397 and 398 (B4-A4 loop) being shared between the three patches (PfK13 sequence numbering; Fig. 3d and Table 2). These positions are usually involved in BTB-BTB interactions in tetrameric or pentameric states of KCTDs^27^ or in BTB-Cullin interactions in some other BTB-containing protein families in monomeric or dimeric states^26^, as in the X-ray structure of the Elongin C-Cullin2 complex (Supplementary Fig. S6)^37^. This suggests that these K13 BTB sites may be involved in some protein-protein interactions.

**Table 2:** Strength of the spatial correlation of *λ* substitution rates in the BTB and KREP domains of K13 and other proteins.

Protein domain	*l*	*σ*	log Bayes factor
K13_BTB-KREP_	5	0.9	30.84
K13_KREP_	3	0.9	20.67
SHKBP1_BTB_	11	1.5	13.27
KCTD17_BTB_	7	1.3	9.69
KEAP1_KREP_	5	1.7	44.06
KLHL2_KREP_	7	1.5	93.54
KLHL3_KREP_	7	1.3	79.47
KLHL12_KREP_	7	1.5	25.18

*l*, strength of the spatial correlation of site-specific substitution rates *λ* over protein tertiary structure, in Å; *σ*, signal standard deviation measuring the variability of *λ* substitution rates across amino acid sites; log Bayes factor, statistical significance of the spatial correlation of site-specific substitution rates *λ*. A log Bayes factor ≥ 8 identifies a significant spatial correlation of site-specific substitutions rates *λ* in protein tertiary structure (conservative cutoff as suggested by FuncPatch’ authors)^33^.

### The KREP domain of K13 exhibits a conserved, rigid, solvent-exposed shallow pocket

**Figure 4:**
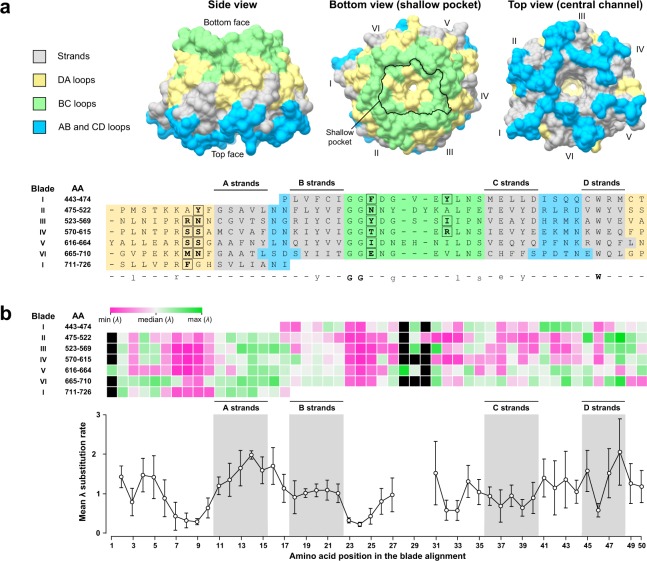
Structural organization of amino acid conservation across K13 KREP. (**a**) Structure-based amino acid alignment of the six blade (or kelch) repeats of PfK13 KREP. The *x axis* shows the position in the structure-based amino acid alignment of the six blades; the left *y axis* shows the amino acid blade number, followed by the first and last amino acid (AA) positions of the corresponding blade. The strands of PfK13 KREP are colored in grey; the AB and CD loops forming the top face of the KREP are colored in cyan; the BC and DA loops architecting the bottom face of the KREP are colored in yellow and green respectively. A consensus sequence of the blades was defined and is shown below the alignment: strict consensus amino acids are shown in bold capital letters and highly conserved amino acids are shown in standard lowercase. A mapping of the different strands and loops onto the three-dimensional structure of PfK13 KREP is shown as surface representation above the structure-based amino acid alignment: side view (*left*), bottom view (*middle*) and top view (*right*). The outline of the shallow pocket surface was delineated with a black line on the bottom view structure (*middle*) and the amino acid sites forming the shallow pocket surface were surrounded and written in bold in the structure-based amino acid alignment. (**b**) Mapping of the site-specific substitution rates *λ* onto the structure-based amino acid alignment of the six blade repeats of PfK13 KREP. *Upper graph*: heat map showing the *λ* substitution rate for each amino acid site. Black boxes correspond to the gaps in the structure-based amino acid alignment. The median of the *λ* substitution rates was first calculated (white) to produce a scale ranging from the lowest (magenta) to the highest (green) site-specific substitution rate *λ*. *Lower graph*: Plot of the mean and 95% confidence interval of the *λ* substitution rates along the structure-based amino acid alignment of the six blades. The positions including one or more gaps were discarded.

In KREP-containing proteins, the KREP domain usually serves as the receptor for substrate(s) further ubiquitinated^24,28^. Before examining the conservation level of the K13 KREP domain, we first re-evaluated its architecture using the resolved 3D structure of PfK13 KREP (PDB code 4YY8, chain A; unpublished results)^38^. The PfK13 KREP structure is composed of six repeats of the kelch motif/blade^5,39^. As expected, each blade is a β-sheet secondary structure involving four twisted antiparallel β-strands (numbered A to D). The innermost A strands line the central channel of the KREP fold whereas the outermost D strands are part of the KREP outside surface. The top face of the domain, containing the central channel opening, is formed by residues from several strands and from AB and CD loops. Although we cannot exclude that the central channel can carry some functions, it was reported to be mainly a structural consequence of kelch motif assembly^40^. The bottom face of PfK13 KREP is composed of residues from the DA and BC loops and contains a shallow pocket, similarly to other KREP structures^24^. Since there is no conventional definition for the shallow pocket delineation in KREP fold, we defined it as the amino acids forming the surface plan of the pocket and protruding out of the plan (n = 19 positions; Figs. 4a and S7).

To characterize the pattern of conservation within the domain, we superimposed the site-specific substitution rates *λ* inferred by FuncPatch onto an amino acid sequence alignment of the six blades (Fig. 4b), custom-produced from a structural alignment of the six blades using PyMOL (Figs. 4a and S8). We found that: *(i)* the conservation level significantly differed between the six blades (*p* = 3.0 × 10^−3^, Kruskal-Wallis *H* test), with blade VI exhibiting the lowest level of conservation (Figs. 4b and S9); *(ii)* loops were surprisingly more conserved than strands (*p* = 5.0 × 10^−3^, Mann-Whitney *U* test; Fig. 4b); *(iii)* the solvent-exposed A and D strands were less conserved than the buried B and C strands (*p* = 1.3 × 10^−6^, Kruskal-Wallis *H* test; Fig. 4b); and (*iv*) the conservation level was the strongest at the blade positions 7–10 (DA loops) and 23–25 (BC loops) (Fig. 4b), which altogether formed the surface and underlying layer of the shallow pocket in the PfK13 KREP tertiary structure (Fig. 4a).

**Figure 5:**
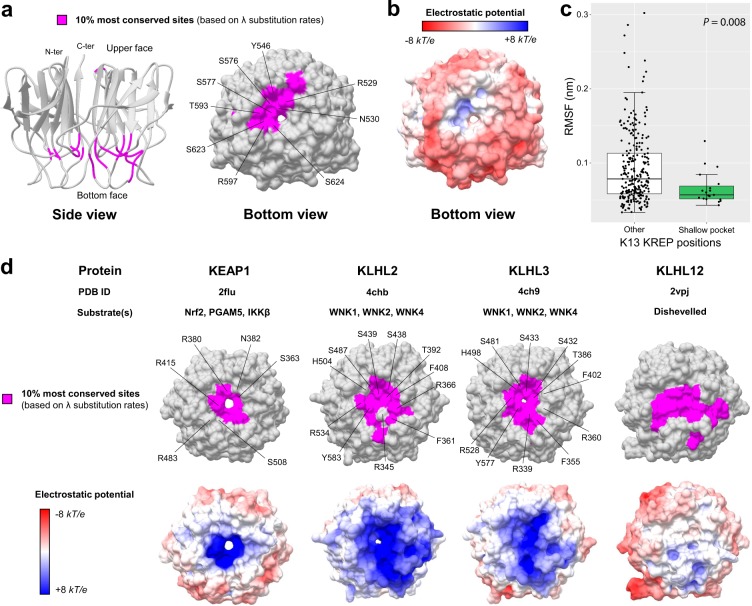
Conservation level and electrostatic potential across the KREP structures of K13 and other BTB-Kelch proteins. (**a**) Location of the 10% most conserved amino acid sites (magenta) on the three-dimensional structure of PfK13 KREP. The conservation level of positions was defined using the site-specific substitution rates *λ* estimated with FuncPatch (Supplementary Dataset S1). The KREP structure is shown from the side view as cartoon (*left* structure) and from the bottom view as surface (*right* structure). The amino acid sites forming the surface of the shallow pocket and belonging to the 10% most conserved sites are labelled. (**b**) Electrostatic surface potential of the PfK13 KREP structure, estimated with the APBS method. Electrostatic potential values are in units of *kT*/*e* at 298 K, on a scale of −8 *kT/e* (red) to +8 *kT/e* (blue). White color indicates a neutral potential. The missing charges were added using the *Add Charge* function implemented in USCF Chimera. (**c**) Box plots showing the distribution of root-mean-square fluctuations (RMSFs; Supplementary Dataset S2) for the PfK13 KREP shallow pocket positions (*shallow pocket* group, green) and the remaining PfK13 KREP positions (*other* group, white). RMSFs were calculated through a molecular dynamics simulation for a duration of 100 ns. Box boundaries represent the first and third quartiles and the length of whiskers correspond to 1.5 times the interquartile range. The difference between groups was evaluated by non-parametric Mann-Whitney *U* test. (**d**) Location of the 10% most conserved amino acid sites and electrostatic potential for KEAP1, KLHL2, KLHL3 and KLHL12 KREP structures. The color code and structure orientation are the same as for PfK13 in panels a and b. For KEAP1, KLHL2 and KLHL3, the key amino acids interacting with their respective protein substrates are labelled. The PDB codes and protein substrates are provided above each KREP structure.

The 10% most conserved of the 284 sites in K13 KREP were all located at the bottom side of the KREP fold, and formed a statistically significant patch (Fig. 5a and Table 2). Importantly, the shallow pocket of K13 KREP (19 positions) was significantly enriched in the 10% most conserved KREP amino acid sites (*p* = 1.6 × 10^−5^, chi-squared test; Fig. 5a, Tables 3 and S3). Of note, the slowly evolving sites predicted by PAML were scattered and did not form a clearly bounded region at the shallow pocket (*p* = 0.704, Fisher’s exact test), and mostly corresponded to the signature residues of KREP proteins (Supplementary Fig. S10). This was somewhat expected since PAML does not consider the statistical information from neighboring sites with similar substitution rates^36^.

**Table 3:** Conservation level of the protein sites constituting the K13 KREP shallow pocket.

AA^*a*^ position	Ref. AA^*a*^ (*P. fal*)	PfK13 mutant^*b*^	Sequence variation across *Apicomplexa* species		*λ*-based KREP rank n, (%)^*c*^
			*Plasmodium* (n = 21)	Non-*Plasmodium* (n = 22)	
451	F	—	F (21)	H (16); C (4); T (2); S(1); N (1)	132 (46.48)
456	Y	—	Y (21)	H (12); I (7); P (2); Y (1)	125 (44.01)
482	Y	S^AFR^	Y (21)	Y (17); H (5)	37 (13.03)
498	N	I^AFR^	N (21)	Q (13); Y (7); H (2)	44 (15.49)
**529**	**R**	**K** ^**AFR**^	**R (21)**	**R (22)**	**1 (0.35)**
**530**	**N**	**K** ^**AFR**^ **/Y** ^**AFR**^	**N (21)**	**N (22)**	**9 (3.17)**
**546**	**Y**	**—**	**Y (21)**	**F (22)**	**13 (4.58)**
551	I	—	I (21)	I (19); V (3)	86 (30.28)
**576**	**S**	**L** ^**AFR**^	**S (21)**	**S (22)**	**3 (1.06)**
**577**	**S**	**P** ^**AFR**^	**S (21)**	**S (22)**	**10 (3.52)**
**593**	**T**	**S** ^**AFR**^	**T (15); A (6)**	**T (22)**	**19 (6.69)**
**597**	**R**	**—**	**R (21)**	**R (22)**	**12 (4.23)**
**623**	**S**	**C** ^**SEA**^	**S (21)**	**S (22)**	**2 (0.70)**
**624**	**S**	**—**	**S (21)**	**A (15); G (7)**	**23 (8.10)**
640	I	V^AFR^	I (21)	I (19); M (2); V(1)	42 (14.79)
671	M	—	M (21)	M (17); L (3); I (1); F (1)	73 (25.70)
672	N	—	N (21)	D (22)	47 (16.55)
688	E	—	E (21)	Q (22)	45 (15.85)
717	F	—	F (21)	Y (19); S (3)	33 (11.62)

^*a*^AA: amino acid position. Ref. AA indicates the wild type amino acid in PfK13. ^*b*^None of the PfK13 mutant positions located at the K13 KREP shallow pocket was reported to confer the ART-R phenotype. All PfK13 mutants presented here have been described in one or two parasite samples from large population surveys^5,8,9,91,92^. AFR (Africa) and SEA (Southeast Asia) refer to the geographic origin of the PfK13 mutant parasite isolate. ^*c*^The rank attributed to the shallow pocket positions was based on the site-specific substitution rates *λ* (Supplementary Dataset S1) estimated for the 284 studied KREP positions using FuncPatch^33^. The lower the rank, the more conserved was the position. Positions in bold are those belonging to the 10% most conserved positions of KREP.

Using the PfK13 KREP structure as reference, we also identified three remarkable features of the conserved patch predicted by FuncPatch. First, it overlapped with a region of the shallow pocket harboring a slightly electropositive surface potential, in contrast to the overall electronegative one of the KREP bottom surface (Fig. 5b). Second, it contained several evolutionarily conserved arginine and serine residues (PfK13 R529, S576, S577, R597 and S623; Fig. 5a and Table 3), which are known to mediate protein-protein interactions in the pocket of other KREP domains^41,42^. And third, the shallow pocket, although being at the domain surface, was more rigid compared to the rest of the KREP structure (*p* = 8.0 × 10^−3^, Mann-Whitney *U* test), as measured by the root-mean-square fluctuation values (RMSF, which measures to what extent a given residue changes its position over time) during a molecular dynamics simulation of 100 nanosecond (ns) at a temperature of 300 K (Fig. 5c; two other replicates produced similar results, data not shown). Altogether, our analyses of K13 KREP revealed that the shallow pocket is extremely conserved, poorly flexible, and differentially charged compared to the remaining KREP bottom face.

### The conserved K13 KREP patch is related to KREP binding activities in well-characterized BTB-Kelch proteins

To evaluate the reliability of FuncPatch to infer conserved functional sites in the context of KREP folds, we studied four other BTB-Kelch proteins found in mammals and which are functionally and structurally well-characterized and involved in E3 ubiquitin ligase complexes (KEAP1, KLHL2, KLHL3 and KLHL12). All these proteins are known to bind substrate proteins through validated binding residues located in their KREP shallow pocket: Nrf2 for KEAP1^42^, WNK for KLHL2 and KLHL3^41^, and Dishevelled for KLHL12^43^. Using large sets of orthologous amino acid sequences (ranging from 129 sequences for KLHL12 to 162 sequences for KLHL2), a statistically significant spatial correlation of site-specific substitution rates *λ* was detected for each KREP fold (Table 2). In each case, the 10% most conserved KREP positions clustered in their respective shallow pocket (highest *p* = 1.4 × 10^−7^ for KEAP1, chi-squared test; Fig. 5d and Supplementary Table S3) and included some critical amino acid residues for substrate binding. The shallow pocket of KEAP1, KLHL2 and KLHL3 KREP structures also showed a markedly electropositive surface potential while that of KLHL12 was more neutral and similar to that of PfK13 (Fig. 5d). Therefore, these results demonstrate the reliability of our structural-evolutionary approach to detect functional sites in a context of KREP folds.

### K13 KREP positions involved in ART-R are not associated with basic structural and long-term evolutionary parameters

**Figure 6:**
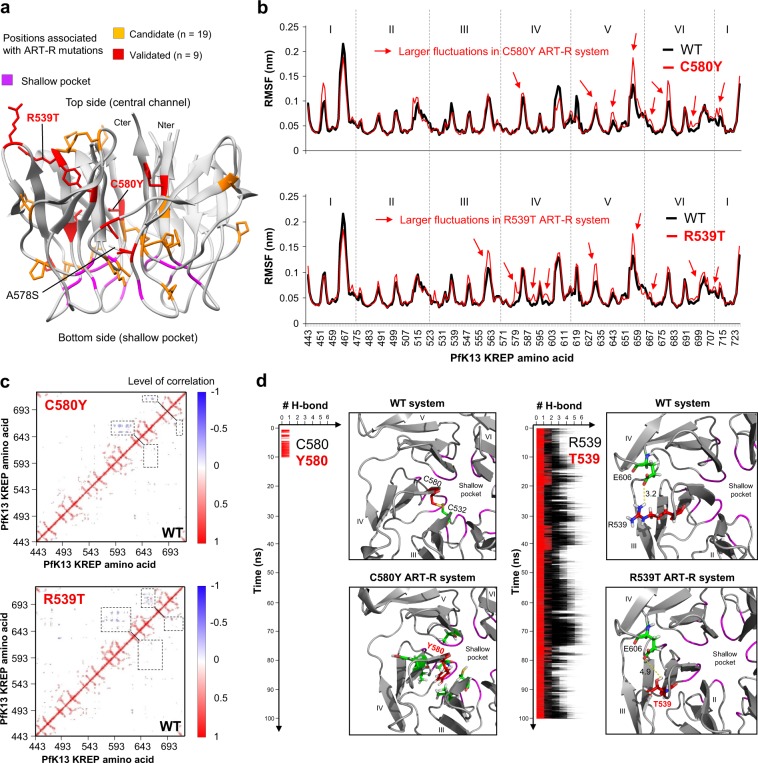
Results of molecular dynamics simulations for WT and ART-R mutant PfK13 KREP structures. Molecular dynamics simulations were carried out on the KREP structure using GROMACS during 100 ns at a temperature of 300 K in an all-atom system. The first ns (0 to 5 ns) correspond to an equilibration phase. (**a**) Mapping of PfK13 ART-R mutations onto the KREP structure. The positions associated with a validated or a candidate ART-R mutation are colored in red and yellow respectively. Validated and candidate ART-R mutations are defined on the basis of the last WHO status report on ART-R^44^. Amino acids forming the shallow pocket are colored in purple. The mutations studied by molecular dynamics simulations are labelled, including the A578S, which is not associated with ART-R. (**b**) Root-mean-square fluctuation (RMSF) values of WT (black) and mutant ART-R (red) PfK13 KREP structures. RMSF per position was calculated on the backbone Cα atoms (excluding the first five ns, corresponding to the equilibration phase). (**c**) Dynamical cross-correlation maps (DCCMs) of WT (*bottom right*) and mutant ART-R (*top left*) systems. In DCCMs, positive correlation for a pair of residues (red) implies that the two residues move in the same direction, while negative correlation (blue) indicates that the two residues move in opposite directions. Dashed boxes correspond to the differential movements between the WT and mutant systems. Maps were generated using Bio3D in R^87^. (**d**) Local impacts of C580Y and R539T ART-R mutations on PfK13 KREP structure. Blade number and location of the shallow pocket are indicated. Inter-atomic distances are expressed in Å.

Several non-synonymous mutations in PfK13 KREP have been reported to confer ART-R to *P. falciparum* parasites from SEA^5–9^. Here, we tested whether these ART-R positions have specific evolutionary or structural properties. Therefore, K13 KREP positions were classified as associated (n = 28) or not (n = 256) with an ART-R mutation, on the basis of the last World Health Organization (WHO) status report on ART-R (listed in Supplementary Table S4)^44^. First, we observed a wide distribution of ART-R positions across the KREP fold (Fig. 6a), but none located at the shallow pocket, although this trend was not statistically confirmed (*p* = 0.23, Fisher’s exact test; 0/19 ART-R mutations for the shallow pocket positions, and 28/265 ART-R mutations for the remaining PfK13 KREP positions). Second, no difference in the inter-species spatially correlated site-specific substitution rates *λ* was observed between the two groups of KREP positions (*p* = 0.96, Mann-Whitney *U* test; Supplementary Fig. S11). Of note, ten positions associated with an ART-R mutation exhibited a substantially higher substitution rate than position 580 – associated with the *pfk13* C580Y allele which has outcompeted other alleles in the East Thailand-Cambodia-Lao PDR-Vietnam region^7^ (Supplementary Table S4). Finally, three structural parameters were also estimated for each position using the WT PfK13 KREP structure^45^: the relative solvent accessibility (RSA) which measures to what extent an amino acid is accessible to the solvent, the side-chain weighted contact number (WCN_sc_) which estimates how densely packed a residue is within the protein tertiary structure, and the RMSF mentioned above (which measures to what extent a given residue changes its position over time). No difference was detected for these structural parameters between the KREP positions associated or not to ART-R (RSA: *p* = 0.44; WCN_sc_: *p* = 0.46; RMSF: *p* = 0.20, Mann-Whitney *U* test; similar results were obtained with other replicates for RMSF; Supplementary Fig. S11). Altogether, these results suggest that K13 KREP positions involved in ART-R are not associated with basic structural and long-term evolutionary parameters.

### PfK13 ART-R mutations cause local structural destabilization of KREP

Next, we tested whether the introduction of amino acid changes that confer ART-R would alter the PfK13 KREP structure, and more specifically the putatively functional shallow pocket. Here, we focused on the ART-R mutations C580Y and R539T because they are the most prevalent and associated with very high level of ART-R respectively. The mutations were introduced individually *in silico*, then the mutant structures were subjected to molecular dynamics simulations during 100 ns (namely C580Y ART-R and R539T ART-R systems). As controls, we studied the WT structure and an ART-sensitive mutant structure (A578S, commonly found in Africa; A578S ART-S system). In all the mutant systems, we did not detect any striking global defect of the KREP structure, as assessed by the total number of hydrogen bonds (H-bonds) in KREP (Supplementary Fig. S12a) and by both the RMSD (Supplementary Fig. S12b) and solvent accessible surface area (Supplementary Fig. S12c) that were averaged across all KREP positions. Also, the electrostatic surface potential of the WT and mutant structures were similar (Supplementary Fig. S12d).

We then looked at individual positions across the molecular dynamics simulations of the WT and mutant systems by examining the spatial fluctuations of KREP backbone Cα atoms (RMSF data). Larger fluctuations of amino acid residues from blades IV, V and VI were observed in the mutant ART-R systems compared to the WT system (Fig. 6b). This contrasts with the A578S ART-S system in which fewer fluctuations were found compared to the WT system (Supplementary Fig. S13a). The fluctuations were similar for the amino acid residues forming the shallow pocket in the mutant systems compared to the WT system (Supplementary Fig. S14a). We further calculated the dynamical cross-correlation maps (DCCMs) which measure for each pair of residues whether they move in the same direction (correlated motions) or in opposite directions (anti-correlated motions), using the backbone Cα atoms along the molecular dynamics simulations. Increased anti-correlated motions were observed in the mutant ART-R systems – especially for R539T – compared to the WT system (Fig. 6c), involving amino acid residues located on blades IV, V and VI, which is consistent with RMSF profiles. No difference in correlated and anti-correlated motions of KREP residues was found between the A578S ART-S and WT systems (Supplementary Fig. S13b).

Finally, we looked at the local environment of the mutant residues. In the C580Y ART-R system, a putative disulfide bridge C532-C580 (blade III-blade IV) was lost (Fig. 6d). In addition, H-bonds involving C580Y (blade IV) with amino acid residues from blades III, IV and V were found only during the first ten ns of the simulation whereas they were not found at all in the WT system (Fig. 6d). The gain and then early, definitive loss of these H-bonds suggested a local rearrangement of the amino acids close to C580Y and that the loss of the disulfide bridge destabilizes the C580Y structure. In the R539T ART-R system, the R539T residue (blade III) was involved in a drastically lower number of H-bonds, including the complete loss of the salt bridge with E606 (blade IV), which was systematically found during the 100 ns simulation in the WT system (Fig. 6d). The loss of an inter-blade H-bond may affect structure stability. In the A578S ART-S system, one new intra-blade H-bond with G591 (blade IV) was recurrently gained and lost during simulations compared to the WT system (Supplementary Fig. S13c), suggesting that it is not critical for the structure stability. Interestingly, none of the tested mutations was found to alter the H-bond network at the shallow pocket (Supplementary Fig. S14b).

Altogether, these results suggest that *pfk13* R539T and C580Y ART-R mutations cause local structural destabilization of KREP rather than directly altering the putative binding region (shallow pocket).

## Discussion

Combining evolutionary and tertiary structure information provides a powerful and efficient way to gain insight into the functionality of protein sites^45^. People usually search for amino acid sites that have evolved more rapidly than expected under a neutral model and interpret them as a signature of adaptive evolution corresponding to a gain of new function(s)^46,47^. Here, we focused on the most slowly evolving, patches of amino acid sites in 3D structure to identify sub-regions of K13 that are likely to play a function conserved during the long time-period of *Apicomplexa* evolution. Detecting highly conserved sites in extremely conserved genes, such as *pfk13*, through population genetics would require a very large sampling of gene sequences^48^, which has not yet been reached for *pfk13*^9^.

In the context of a sustained and intense purifying selection operating in all annotated domains of K13, our inter-species analysis of K13 sequence evolution coupled to the BTB-KREP tertiary structure of PfK13 identified two patches of particularly slowly evolving sites. The most striking conserved patch is found in the K13 KREP domain and is formed by specific positions from inter- and intra-blade loops. This conserved patch largely overlaps with a solvent-exposed, shallow pocket located at the bottom face of the KREP structure (opposite to the face with the central channel entry). Extreme conservation of amino acid sites could be due to constraints linked to the protein structure – for proper folding and stability – or function^45^. Several lines of evidence suggest that the extremely conserved shallow pocket of K13 contains functional sites. First, the pocket is highly enriched in conserved positions, whereas solvent-exposed sites in proteins are usually evolutionarily less conserved than buried ones^45^. Second, a similar shallow pocket is found in the KREP domain of well-characterized BTB-Kelch proteins (KEAP1, KLHL2, KLHL3 and KLHL12) which act as E3 ubiquitin ligase substrate adaptors. In those proteins structurally related to K13, the pocket directly mediates KREP’s binding activities^24,41,42^ and was also predicted as a conserved patch in our analyses. The KREP shallow pockets of K13 and other BTB-Kelch were found to share interesting properties: they display a differential electrostatic surface potential, as compared to the rest of the KREP bottom face, and they are rich in highly conserved arginine and serine residues (PfK13 R529, R597, S576, S577 and S623; Table 3). In KEAP1, KLHL2 and KLHL3, the corresponding conserved arginine and serine residues directly bind acidic peptides derived from their substrates, the transcription factor Nrf2 and kinases WNK respectively^41,42^. The electrostatic surface potential of the shallow pocket of K13 is slightly positive, therefore the K13 substrate molecule(s) may also harbor an electronegative binding motif. Altogether, these results indicate that the shallow pocket of K13 KREP exhibits several properties of a conserved, binding surface and we speculate that it may be critical for the recognition of K13 substrate molecule(s). We propose that conserved arginine and serine residues are putative candidate binding sites in the shallow pocket of PfK13, a hypothesis that could be tested by biochemical experiments using recombinant K13 KREP domains. In *P. falciparum*, PfPI3K is a likely partner candidate as it is immunoprecipitated with full-length PfK13, and its ubiquitination and proteasomal degradation are altered by the PfK13 C580Y mutation on the KREP domain^17^. Another candidate may be the PK4 kinase which phosphorylates eIF2α, a key mediator of translation-mediated latency involved in ART-R^15^.

The *pfk13*-mediated ART-R mechanism in *P. falciparum* is related to the binding activity of PfK13^17^, presumably through the KREP domain since ART-R mutations cluster in this PfK13 domain^5,7,8^. In KEAP1, cancer-related missense mutations in the KREP domain were reported to be pathogenic through diverse molecular effects such as reduced binding of Nrf2 (mainly mutations at Nrf2 binding sites in the shallow pocket) and decreased protein stability or abundance (mutations outside the shallow pocket)^49–51^. In PfK13, it is interesting to note that the 28 validated or candidate *pfk13* ART-R alleles are largely distributed across the PfK13 KREP structure and are all located outside the shallow pocket (Fig. 6a): some validated ART-R alleles have a preferential localization at positions proximal to the A and B strands (F446I, Y493H, R539T, I543T, C580Y)^9^ and some other candidates are found in the underlayer of the pocket. In addition, polymorphisms which have an uncharacterized phenotype were reported at 9 out of the 19 positions forming the shallow pocket, but found in only one or two parasite samples from very large population surveys (Table 3). Therefore, one may speculate that amino acid changes at the shallow pocket positions, that we predict to be directly involved in substrate binding, are too functionally damaging to provide a long-term competitive advantage. Rather, many *pfk13* ART-R alleles may alter other properties of PfK13 such as its abundance, for example through altered protein synthesis or folding or stability. In this scenario, a lesser abundance of mutant – but still functional – PfK13 protein would result in decreased PfK13 cellular activity. In addition to the location of ART-R mutations mentioned above, several data support this *stability* scenario. First, the C580Y ART-R mutation leads to an approximately 2-fold lower abundance of PfK13 protein, as measured by quantitative dimethyl-based proteomics analysis of several strains derived from clinical ART-resistant Cambodian parasites^19^ (note that a different result was reported for two African isogenic strains using western blot analysis^20^). Second, several mutations in the KREP domain of the BTB-Kelch protein Gigaxonin, which cause giant axonal neuropathy in humans^52,53^, were shown to decrease Gigaxonin protein abundance through altered stability, as evidenced by pulse chase experiments^53^. Interestingly, the Gigaxonin mutation C464Y, which is located in the KREP central channel such as C580Y in PfK13, was identified in a compound heterozygous patient and was also associated with a decreased Gigaxonin protein abundance. Third, molecular dynamics simulations on the BTB-Kelch KLHL3 protein revealed that some pathogenic mutations localized in the central channel of KREP (such as C580Y in PfK13), have no effect on KREP-ligand interaction^54^. And finally, the molecular dynamics simulations we carried out on PfK13 R539T and C580Y ART-R KREP structures (at a temperature of 300 K during 100 ns) showed that R539T and C580Y mutations cause structural destabilization on blades IV, V and VI, at amino acid positions that are not located at the shallow pocket.

BTB appeared here as the most conserved domain of K13 over *Apicomplexa* evolution, and therefore likely carries critical activities. One surprising result of our study is that it most resembles the BTB domain of the KCTD protein family in primary sequence, tertiary structure and short domain size. KCTD proteins are often found in E3 ubiquitin ligase complexes, such as BTB-Kelch proteins, but lack a KREP domain^27^. The shortened BTB domain of KCTDs can mediate protein oligomerization^26,27,55^, consistent with the dimer observed in the solved BTB-KREP crystal structures of PfK13 (PDB codes 4YY8 and 4ZGC)^38,56^. The BTB domain of K13 harbors a predicted, functional patch – located at the B2-B3 and B4-A4 loops and at the A4 helix – that overlaps with the patch we found on the BTB domain of KCTDs. In KCTDs, these sites make BTB-BTB contacts in tetrameric or pentameric assemblies when BTB is solved as an isolated domain^27^. However, the PfK13 BTB-KREP structure forms a dimer and none of the highly conserved K13 BTB positions makes BTB-BTB contacts (PDB codes 4YY8 and 4ZGC)^38,56^. In several solved BTB complexes, amino acids of the B4-A4 loop (corresponding to positions 397–399 in PfK13) are exposed at the BTB-Cullin binding interface^26^. These discrepancies in the role of the predicted BTB conserved patch could be due to the fact that additional domains (such as KREP) or partner proteins might constraint the folding of the BTB domain into oligomers or complexes. Altogether, data from the literature however support that the BTB predicted patch of K13 may mediate protein-protein interactions, possibly with a Cullin protein.

Interestingly, K13 also contains a highly conserved CCC domain which therefore likely carries critical activities. Consistent with this hypothesis, *pfk13* R239Q, E252Q and D281V alleles located in CCC confer ART-R, although at a moderate level compared to KREP mutations^3,7,57^. Coiled-coils are ubiquitous protein-protein interaction domains composed of two or more α-helices coiling together^58^. A CCC domain was reported in a few KREP-containing proteins (including some KCTDs) involved in cell morphogenesis but these CCC have a different domain organization than the one of K13^39^. The CCC of K13 may participate in K13 oligomerization and/or serve as a binding interface with other molecules^59^.

In conclusion, by comparative structural and evolutionary analyses, we identified the shallow pocket of the K13 KREP domain as a likely candidate surface for binding substrate molecule(s). Importantly, we observed that C580Y and R539T ART-R mutations cause local structural destabilization of the KREP structure rather than directly altering the shallow pocket. We also detected in the K13 BTB domain a conserved patch of sites that are involved in protein-protein interactions in known BTB-Cullin and BTB-BTB complexes. Efforts should now focus on the validation of the binding properties of the K13 KREP shallow pocket and identify its binding partner(s). This may help to clarify the structure-function relationship in K13.

## Materials and Methods

### Collection of *k13* orthologous sequences from genomic databases

The amino acid sequence of PfK13 (PlasmoDB code PF3D7_1343700) was queried against the specialized eukaryotic pathogen database EuPathDB (release 33)^60^ and the NCBI non-redundant protein database using blastp and tblastn searches (BLOSUM62 scoring matrix)^61^. A protein was considered as a likely orthologous sequence if the sequence identity was ≥30% and the e-value below the 10^−3^ cutoff. Forty-three K13 sequences – and corresponding *k13* cDNA sequences – were retrieved from distinct *Apicomplexa* species including 21 *Plasmodium* species. A detailed bioinformatics analysis was performed on each protein sequence to confirm the presence of the three annotated domains of K13 (CCC, BTB and KREP) using InterPro^62^.

### *k13* codon sequence alignment

Considering the greater divergence of coding nucleotide sequences as compared to protein sequences due to the genetic code redundancy, a K13 protein sequence alignment was first generated using MAFFT version 7 (E-INS-I strategy with BLOSUM62 scoring matrix, gap opening penalty 2.0 and offset 0.1)^63^. The output alignment was visually inspected and manually edited with BioEdit v7.2.5^64^. The positions containing gaps in at least 30% of all sequences were removed for further evolutionary analyses, as suggested by PAML’ author^34^. Then, the *k13* nucleotide sequence alignment was generated with PAL2NAL^65^ using the cleaned K13 amino acid alignment as template.

### Phylogenetic analysis of *k13*

The phylogenetic relationships of *k13* nucleotide sequences were inferred using the maximum-likelihood method implemented in PhyML v3.0^66^, after determining the best-fitting nucleotide substitution model using the Smart Model Selection (SMS) package^67^. A general time-reversible model with optimized equilibrium frequencies, gamma distributed among-site rate variation and estimated proportion of invariable sites (GTR + *G* + *I*) was used, as selected by the Akaike Information Criterion. The nearest neighbor interchange approach was chosen for tree improving, and branch supports were estimated using the approximate likelihood ratio aLRT SH-like method^68^.

### Molecular evolutionary analysis of *k13*

To investigate the evolutionary regime that has shaped the *k13* protein-coding DNA sequence during species evolution, we analyzed the non-synonymous (*d*_N_) to synonymous (*d*_S_) substitution rate ratio *ω* (=*d*_N_*/d*_S_), estimated by maximum-likelihood using the codeml tool from PAML v.4.8^34,69^. *ω* provides a sensitive measure of selective pressure at the amino acid level by comparing substitution rates with statistical distribution and considering the phylogenetic tree topology. Typically, *ω* <1 indicates purifying selection, while *ω* = 1 and *ω* >1 indicate neutral evolution and positive selection respectively.

The variation of *ω* among lineages and codon sites was evaluated using codon models free-ratio (FR), M0, M1a, M2a, M3, M7 and M8^70,71^ and statistically compared using likelihood ratio tests (LRTs)^72^. The details of each model and PAML interpretations can be found in Supplementary Methods S1.

In addition to *k13*, four other five- or six-bladed KREP protein-coding sequences were considered to compare their *ω* with those estimated for the whole *Plasmodium* proteome. We used the *ω* values previously estimated by Jeffares *et al*.^35^ with PAML under the one-ratio model for each of the 3,256 orthologous protein-coding genes among six *Plasmodium* species: *P. falciparum*, *P. berghei*, *P. chabaudi*, *P. vivax*, *P. yoelii* and *P. knowlesi*. A full description of the procedure is presented in the original paper^35^.

### Inferring site-specific substitution rates considering their spatial correlation in the K13 BTB-KREP tertiary structure

Most methods – including PAML – assume that site-specific substitution rates are independently distributed across sites^73^. However, it is widely acknowledged that amino acids located close to each other in protein tertiary structures are more likely to carry out similar functions, suggesting a site interdependence in amino acid sequence evolution attributed to tertiary structure^73,74^. Consequently, the substitution rate at the protein level (named *λ* in this study) was inferred using the FuncPatch server^33^. FuncPatch requires an amino acid sequence alignment, a phylogenetic tree and a protein tertiary structure to estimate the conservation level during species evolution and the characteristic length scale (in Å) of spatially correlated site-specific substitution rates *λ*. We used the X-ray structure at 1.81 Å resolution of WT PfK13 BTB-KREP as the reference structure which does not contain the conserved CCC domain (PDB code 4YY8, chain A)^38^. Beforehand, a Ramachandran analysis was performed to validate the quality of the structure using MolProbity^75^: 96.9% and 3.1% of the amino acids were in favored and allowed regions, respectively, and there were no outliers. FuncPatch only accepts monomeric proteins as input whereas BTB-KREP of PfK13 dimerizes in crystal structure. To take into account the dimeric organization of PfK13, its tertiary structure was edited using customized python scripts (Python v2.7.13) in order to merge the two monomers (chains A and B) and the K13 sequence was duplicated in the K13 protein sequence alignment. The analysis was also done using either one of the other monomeric BTB-KREP tertiary structure and also using a disulfide-bonded version of PfK13 BTB-KREP (PDB code 4ZGC, chain A)^56^. All these control analyses yielded similar results (data not shown) as the one presented here. The spatial correlation of the site-specific substitution rates *λ* in the K13 tertiary structure was tested using a Bayesian model comparison, where a null model (model 0), in which no spatial correlation of site-specific substitution rates *λ* is present, is compared to the alternative model (model 1). As suggested by FuncPatch’ authors, the spatial correlation was considered as significant if the estimated log Bayes factor (model 1 versus model 0) was larger than 8 in the dataset (conservative cutoff)^33^.

### Delineation of K13 KREP blades and secondary structures

The KREP domain of PfK13 is composed of six blades (or kelch motifs) having slightly different amino acid lengths. To get an accurate blade alignment at the primary amino acid sequence level, we first sought to align the six blade structures. The PDB KREP structure was obtained from the PfK13 BTB-KREP structure (PDB code 4YY8, chain A)^38^ and was then divided into six parts, each one containing the atomic coordinates of one blade. The six blade structures were then aligned by minimizing the RMSD of atomic positions using the *align* function in PyMOL Molecular Graphics System (Schrödinger, LLC) so as to identify the amino acids from the six blades that are located at exactly the same blade position. This structure alignment was then used to align the six blades at the primary amino acid sequence level. The delineation of the strands and loops was obtained directly from the PDB file (PDB code 4YY8, chain A)^38^.

### Structural evolutionary analysis of the BTB and KREP domains in other BTB- and KREP-containing proteins

To characterize the BTB domain of K13, we arbitrarily retrieved some members belonging to the main BTB-containing protein families (BTB-ZF, BTB-Kelch, RhoBTB, BTB-NPH3, MATH-BTB, KCTD, KCNA and SKP1 and Elongin C proteins; full list provided in Supplementary Table S5). A multiple protein alignment was generated using MAFFT version 7 (default parameters)^63^ and was then manually edited with BioEdit v7.2.5^64^ to retain only the region referring to the BTB core fold. The phylogenetic relationships were inferred with the PhyML program^66^ using the best-fitting protein substitution model as determined by the SMS package^67^. The model of K13-Cullin2 complex was obtained by a structural alignment using the *align* function in PyMOL (Schrödinger, LLC) of the K13 BTB domain (PDB code 4YY8)^38^ with Elongin C using the X-ray structure at 3.2 Å resolution of Elongin C-Cullin2 complex (PDB code 4WQO)^37^.

For further comparisons with the K13 BTB and KREP domains, site-specific substitution rates *λ* were inferred with FuncPatch for the BTB and KREP domains of several mammalian KCTD and BTB-Kelch proteins, respectively. In the present study, the proteins were selected on the basis of their sequence homology with K13, the availability of a solved 3D structure, and their known implication in a Cullin-RING E3 ligase complex as suspected for K13. In addition, only well-characterized ligand-binding function and the presence of a six-bladed KREP structure similar to the one of K13 were considered for BTB-Kelch proteins. After a careful review of the literature, we selected two KCTD proteins: SHKBP1 (UniProt code Q8TBC3) which regulates the epidermal growth factor receptor (EGFR) signaling pathway^76^; and KCTD17 (Q8N5Z5) which mediates the ubiquitination and proteasomal degradation of the ciliogenesis down-regulation TCHP protein^77^. Considering BTB-Kelch proteins, we focused on: KEAP1 (Q14145) which interacts with its client protein Nrf2 for the induction of cytoprotective responses to oxidative stress^42^; KLHL2 (O95198) and KLHL3 (Q9UH77) which both participate in the ubiquitination and degradation of WNK substrates regulating blood pressure^41^; and KLHL12 (Q53G59) which negatively regulates the WNT-beta-catenin pathway through the degradation of Dishevelled proteins^43^. The building of datasets is described in Supplementary Method S2, and the full list of orthologous sequences used for each mammalian KCTD and BTB-Kelch protein is provided in Supplementary Table S6. The phylogenetic relationships were inferred using PhyML^66^ after determining the best-fitting protein substitution model with the SMS package^67^. The 3D structures of KCTD BTB and BTB-Kelch KREP domains were retrieved from the PDBsum database under the following accession numbers: 4CRH for SHKBP1 (resolution: 1.72 Å)^27^, 5A6R for KCTD17 (resolution: 2.85 Å)^27^, 2FLU for KEAP1 (resolution: 1.50 Å, in complex with a Nrf2 peptide)^78^, 4CHB for KLHL2 (resolution: 1.56 Å, in complex with a WNK4 peptide)^41^, 4CH9 for KLHL3 (resolution: 1.84 Å, in complex with a WNK4 peptide)^41^ and 2VPJ for KLHL12 (resolution: 1.85 Å)^24^. Beforehand, the quality of each structure was validated using MolProbity^75^: none of the structures had amino acids identified as outliers, and approximately 98% of the amino acids of each structure were in favored regions.

### Molecular dynamics simulations and evaluation of structural properties

Molecular dynamics simulations were carried out on the KREP domain of PfK13 containing the C532-C580 disulfide bond (PDB code 4ZGC, chain A)^56^ using GROMACS v. 5.0.7^79^ for a duration of 100 ns at a temperature of 300 K. Although the C532-C580 disulfide bond is not present in the second resolved structure (PDB code 4YY8)^38^, the two thiol side-chains face each other in the 4YY8 structure and the C532-C580 disulfide bond is recommended as assessed by the PROPKA program^80^. Because no experimentally mutant PfK13 structures are yet determined, we generated mutant PfK13 KREP structures by individually introducing *in silico* the mutations R539T, C580Y or A578S: we used the *swapaa* function of UCSF Chimera to substitute the WT residue with the most probable rotameric conformation of the mutant residue^81^. Full parameters and options of the molecular dynamics simulations can be found in Supplementary Method S3.

Amino acid flexibility was investigated by calculating root-mean-square fluctuations (RMSFs) on side-chain residues using the inbuilt *rmsf* function in GROMACS. The number of distinct H-bonds formed within the protein during simulations was counted when the donor-acceptor distance is ≤3.5 Å and donor-hydrogen-acceptor angle ≤30°, using the *hbond* function in GROMACS. The electrostatic surface potential of each KREP structure was calculated using the Adaptive Poisson-Boltzmann Solver (APBS) method^82^. Beforehand, the required pqr input files were prepared using PDB2PQR v.2.1.1^83^. The missing charges were added using the *Add Charge* function implemented in USCF Chimera^81^. A grid-based method was used to solve the linearized Poisson-Boltzmann equation at 298 K, with solute (protein) and solvent dielectric constant values fixed at 2 and 78.5, respectively. The contact surface selection was mapped using a radius of 1.4 Å in a scale of −8 *kT/e* to +8 *kT/e*. The relative solvent accessibility (RSA), which quantifies the extent to which a given residue comes into contact with solvent, was estimated as its surface area accessible to water using the DSSP program^84^, then normalized with the maximal accessible surface area of each amino acid^85^. The side-chain weighted contact number (WCN_sc_) of each amino acid, which quantifies the extent to which a given residue comes into contact with other residues in the protein (or packing density), was calculated using a customized python script provided by Sydykova and colleagues^86^. Correlative motions of residues (based on Cα atoms) and correlation degrees were calculated through dynamical cross-correlation maps (DCCMs). A positive correlation (>0) means the pair of residues move in the same direction; a negative correlation (<0) indicates that the pair of residues move in opposite directions. DCCMs were produced using the Bio3D library implemented in R^87^. Beforehand, GROMACS trajectories (in.xtc format) were converted into binary trajectories (.dcd) using the *catdcd* function from VMD^88^, as required by Bio3D.

All structural properties were assessed using the aforementioned PDB files.

### Structure visualization

All molecular drawings were generated using the UCSF Chimera software^81^ or PyMOL (Schrödinger, LLC).

## Supplementary information

Supplementary Figures

Supplementary Tables

Supplementary Methods

Supplementary Dataset 1

Supplementary Dataset 2
