# Supplementary material for: Comparative structural and evolutionary analyses predict functional sites in the artemisinin resistance malaria protein K13
Source: Sci Rep. 2019 Jul 23;9:10675. doi: 10.1038/s41598-019-47034-6 (PMC6650413; doi:10.1038/s41598-019-47034-6)
Supplement: Supplementary file 1 — Supplementary Figures [file 41598_2019_47034_MOESM1_ESM.pdf]

Supplementary Figures

This supplemental file has been provided by the authors to give readers additional information about their work.

Supplement to: **Comparative structural and evolutionary analyses predict functional sites in the artemisinin resistance malaria protein K13**

By Romain Coppée, Daniel C. Jeffares, Maria A. Miteva, Audrey Sabbagh, Jérôme Clain

Figure S1 ………………………………………………………………………………………… 5

Figure S2 ………………………………………………………………………………………… 6

Figure S3 ………………………………………………………………………………………… 7

Figure S4 ………………………………………………………………………………………… 8

Figure S5 ………………………………………………………………………………………… 9

Figure S6 ………………………………………………………………………………………… 10

Figure S7 ………………………………………………………………………………………… 11

Figure S8 ………………………………………………………………………………………… 12

Figure S9 ………………………………………………………………………………………… 13

Figure S10 ……………………………………………………………………………………….. 14

Figure S11 ……………………………………………………………………………………….. 15

Figure S12 ……………………………………………………………………………………….. 16

Figure S13 ……………………………………………………………………………………….. 18

Figure S14 ……………………………………………………………………………………….. 19

Note 1 : Page number is based on figure titles.

Note 2 : Figure S1 is subdivided in multiple pages.

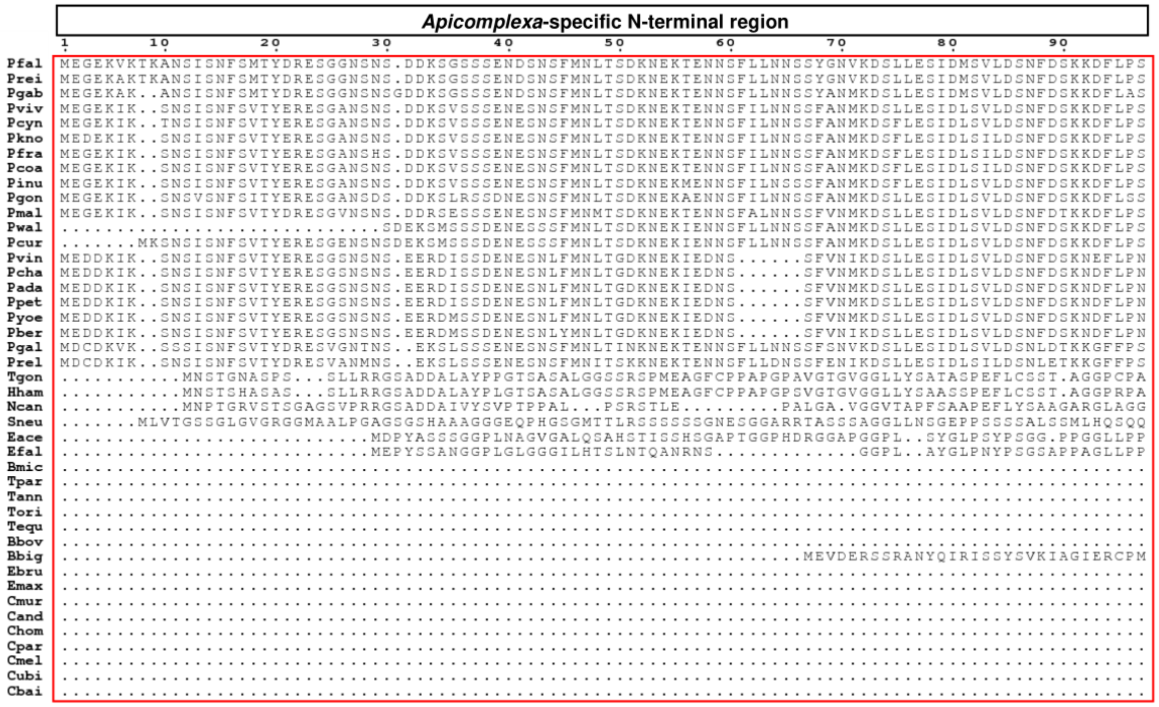
Apicomplexa-specific N-terminal region
Pfal
Prei
Pgab
Pviv
Pcyn
Pkno
Pfra
Pcoa
Pinu
Pgon
Pmal
Pwal
Pcur
Pvin
Pcha
Pada
Ppet
Pyoe
Pber
Pgal
Prel
Tgon
Hham
Ncan
Sneu
Eace
Efal
Bmic
Tpar
Tann
Tori
Tequ
Bbov
Bbig
Ebru
Emax
Cmur
Cand
Chom
Cpar
Cmel
Cubi
Cbai

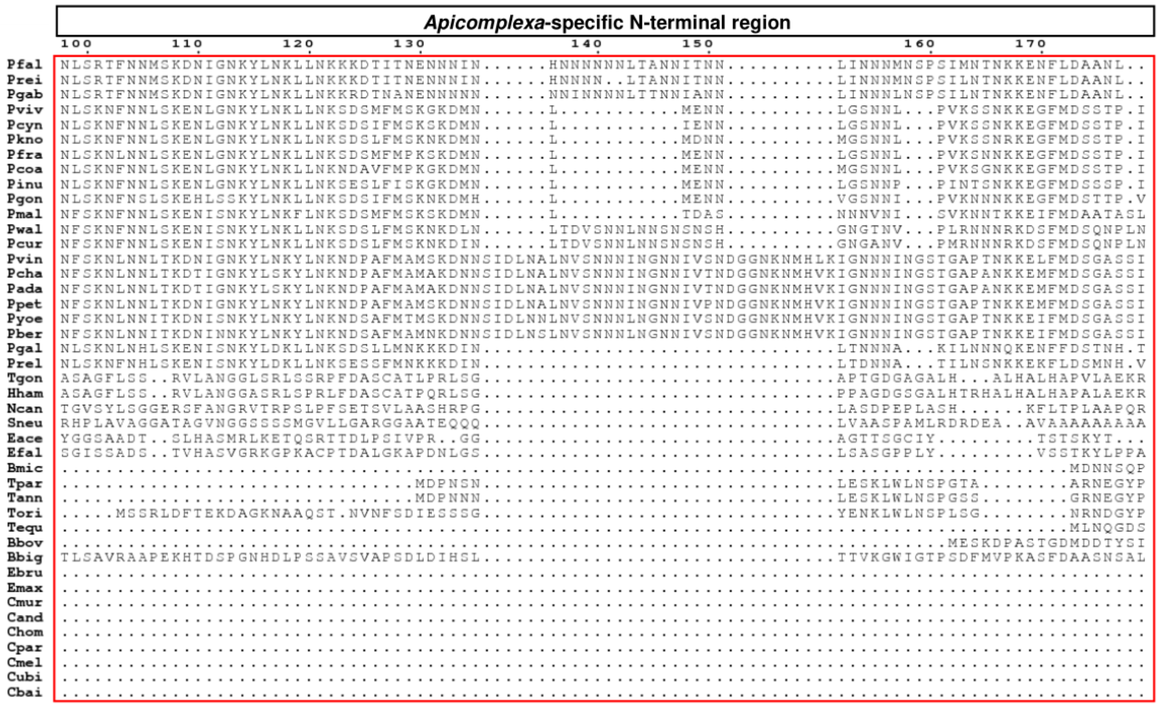
Apicomplexa-specific N-terminal region
Pfal
Prei
Pgab
Pviv
Pcyn
Pkno
Pfra
Pcoa
Pinu
Pgon
Pmal
Pwal
Pcur
Pvin
Pcha
Pada
Ppet
Pyoe
Pber
Pgal
Prel
Tgon
Hham
Ncan
Sneu
Eace
Efal
Bmic
Tpar
Tann
Tori
Tequ
Bbov
Bbig
Ebru
Emax
Cmur
Cand
Chom
Cpar
Cmel
Cubi
Cbai

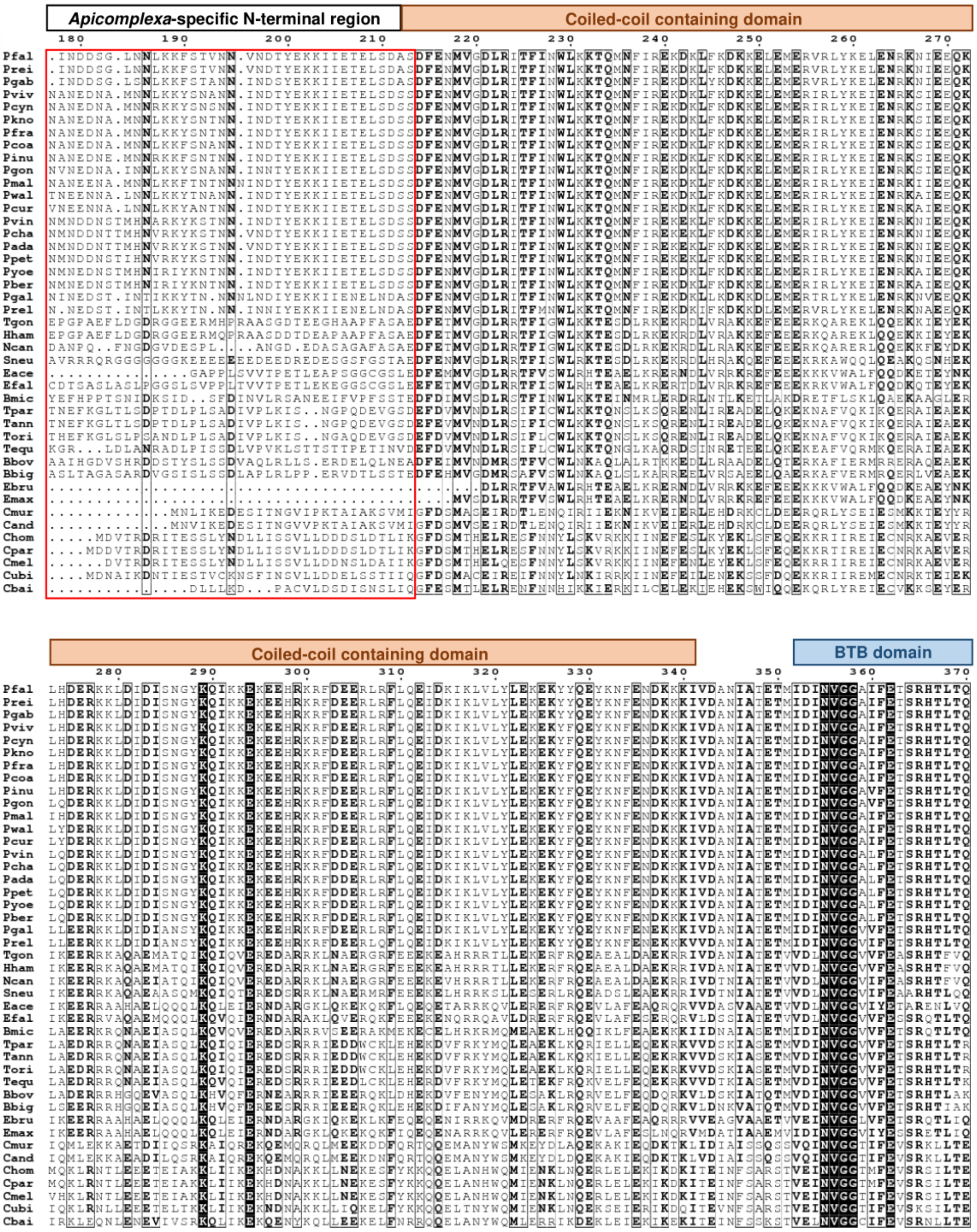
Apicomplexa-specific N-terminal region
Coiled-coil containing domain
Coiled-coil containing domain
BTB domain

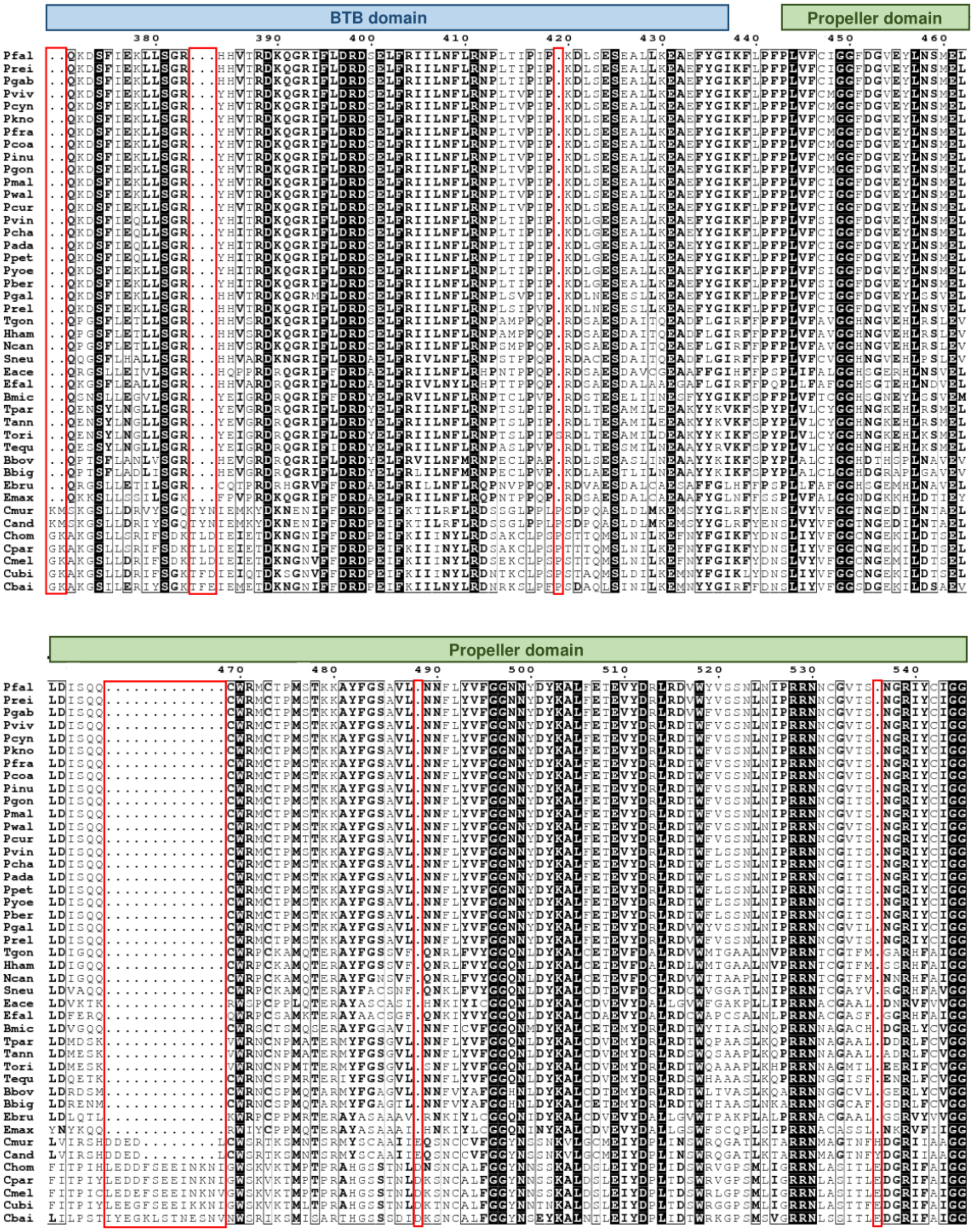
BTB domain
Propeller domain
Propeller domain

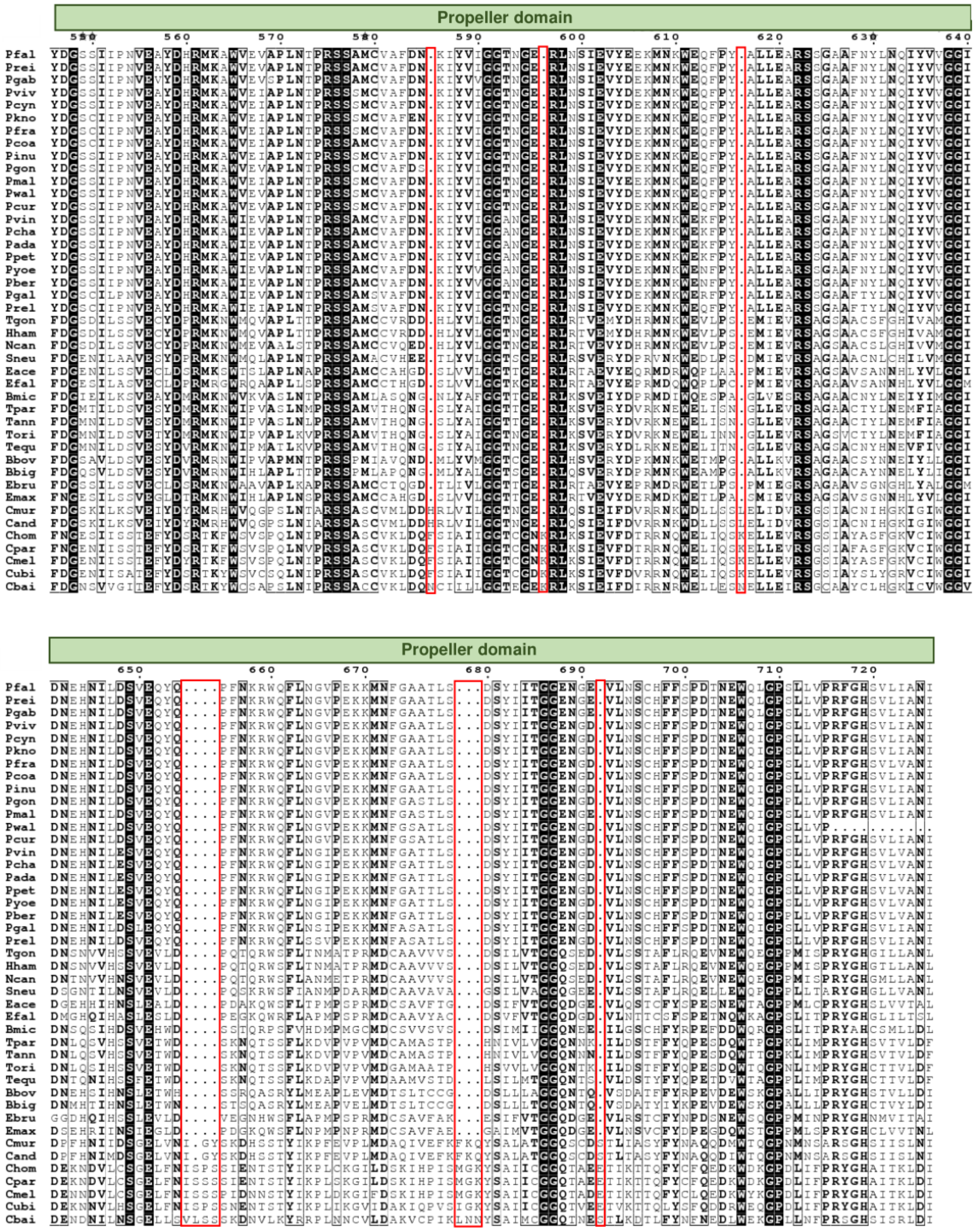

Figure S1. K13 multiple amino acid sequence alignment.

The K13 multiple sequence alignment was performed using Mafft version 7. Strictly conserved positions are written in white letters and shaded in black, and highly conserved positions are boxed with black lines and written in write, bold letters (the level of conservation is based on BLOSUM62 scoring matrix). A four-letter code is used for the name of species (full species names are provided in Supplementary Table S1). The regions of the alignment with highly divergent positions, as well as positions containing gaps in at least 30% of all sequences, were manually removed (red boxes). The multiple alignment was displayed with the ESPript 3.0 server. The annotated domains of K13 (Coiled-coil containing, BTB and propeller (or KREP)) are shown above the K13 multiple sequence alignment.

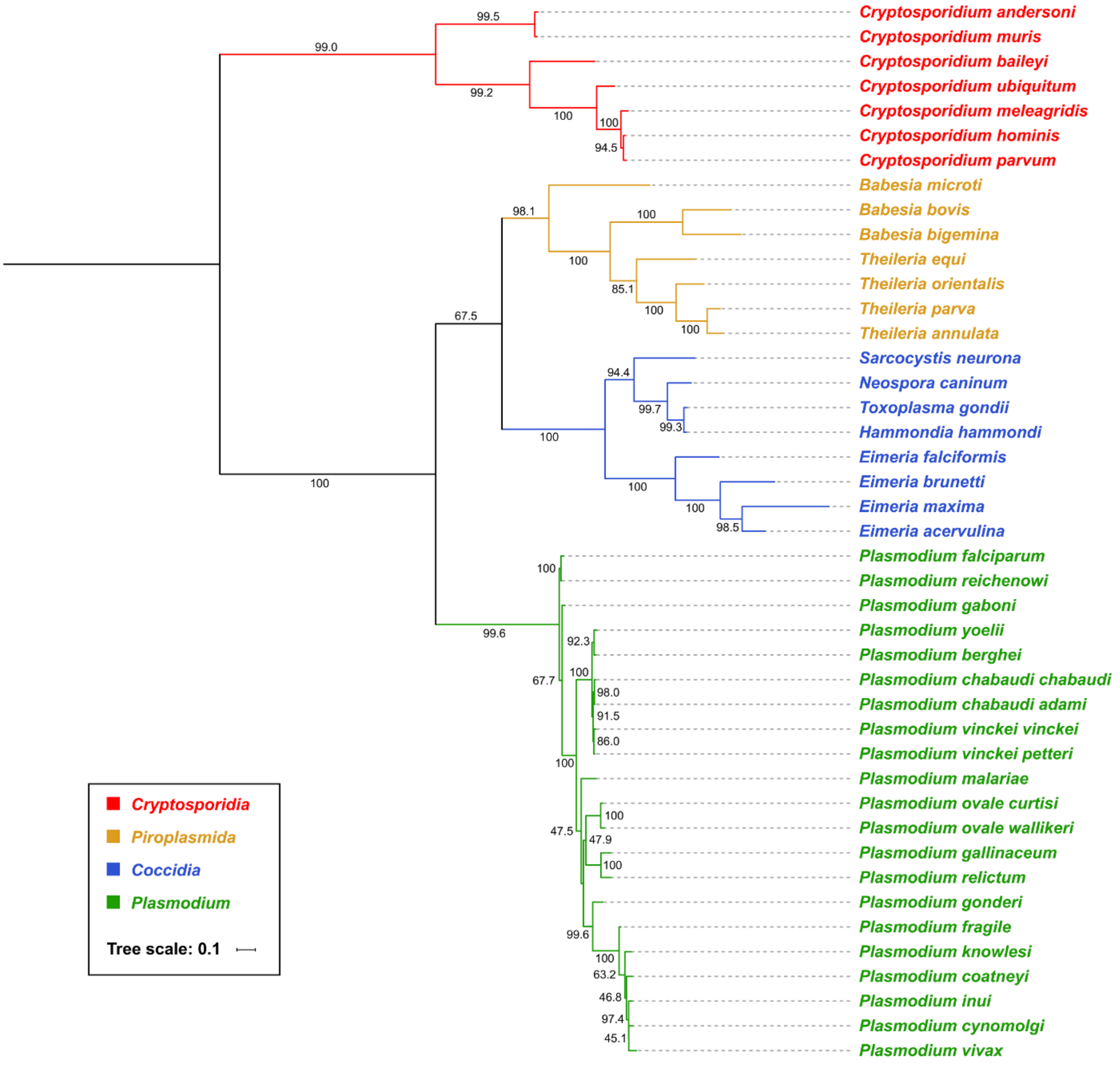

Figure S2. Maximum-likelihood phylogenetic tree of *k13* orthologous protein-coding sequences. All bootstrap values are shown as percentages at nodes using the approximate likelihood-ratio aLRT SH-like method. Colors are associated with the different *genera* or *phyla*: *Plasmodium*, green; *Cryptosporidia*, red; *Coccidia*; blue; and *Piroplasmida*, orange. *Cryptosporidia* was used as outgroup to root the tree. *Hematozoa* should include both *Plasmodium* and *Piroplasmida* species.

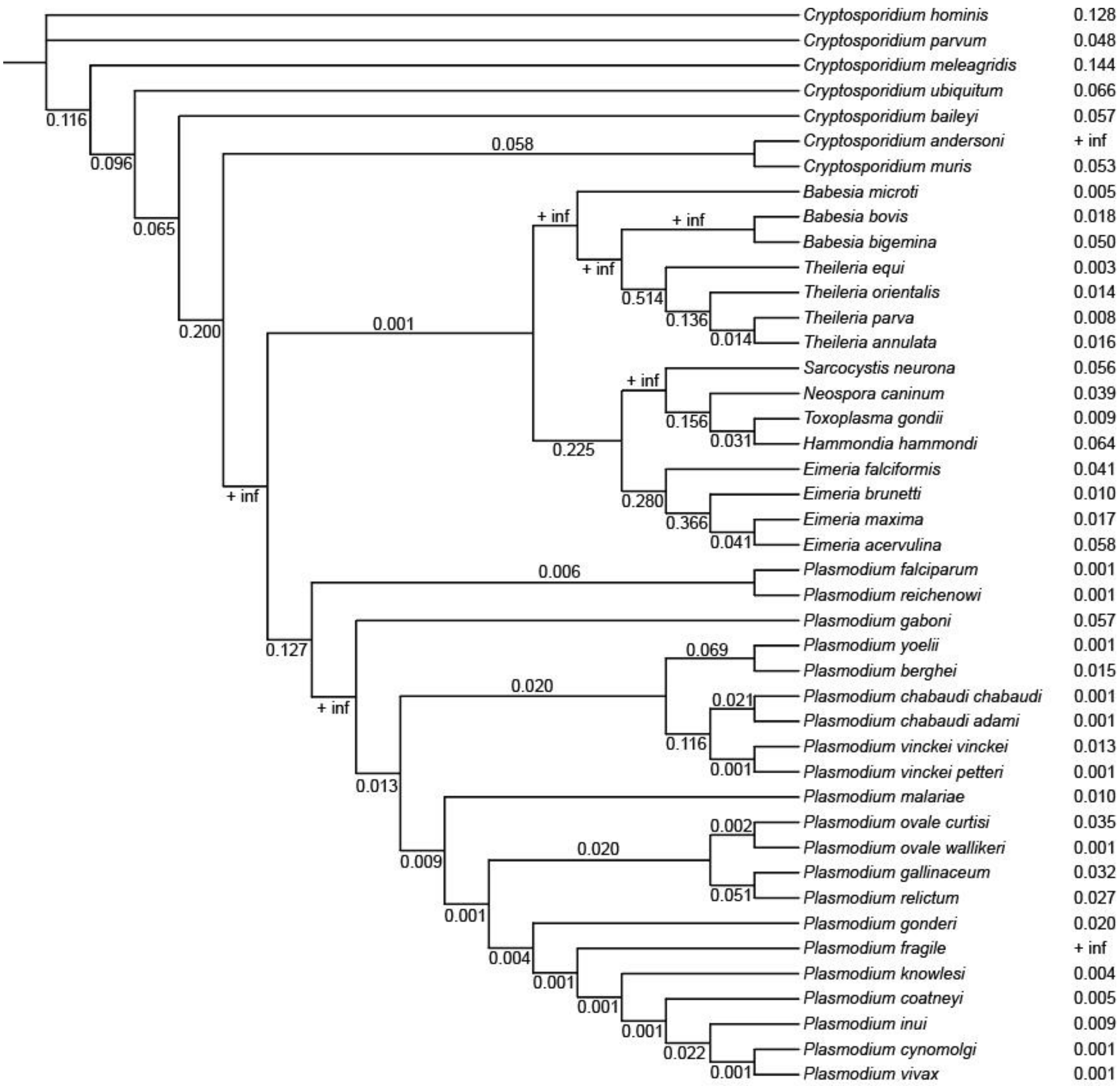

Figure S3. Branch-specific *ω* values estimated with PAML under the free-ratio (FR) model for the *k13* gene.

ω estimates with "+ inf" values for which the estimation of d_S was equal to 0.0 (*i.e.* no silent substitutions were observed) are designated as extremely low. They are therefore not indicative of a positive selection episode. Branch lengths were ignored for ease of representation.

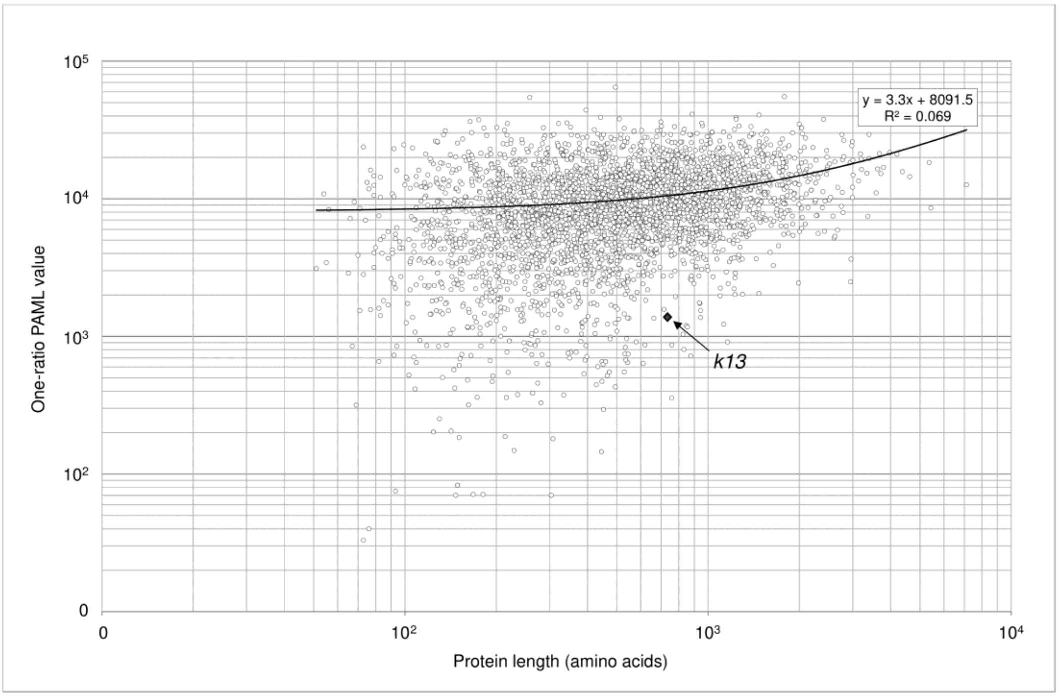

Figure S4. Positive correlation between protein length and one-ratio PAML value for the *Plasmodium* protein-coding genes.

The one-ratio PAML values were multiplied by 10^5 and then plotted using a logarithmic scale. K13 is shown as a full-black diamond.

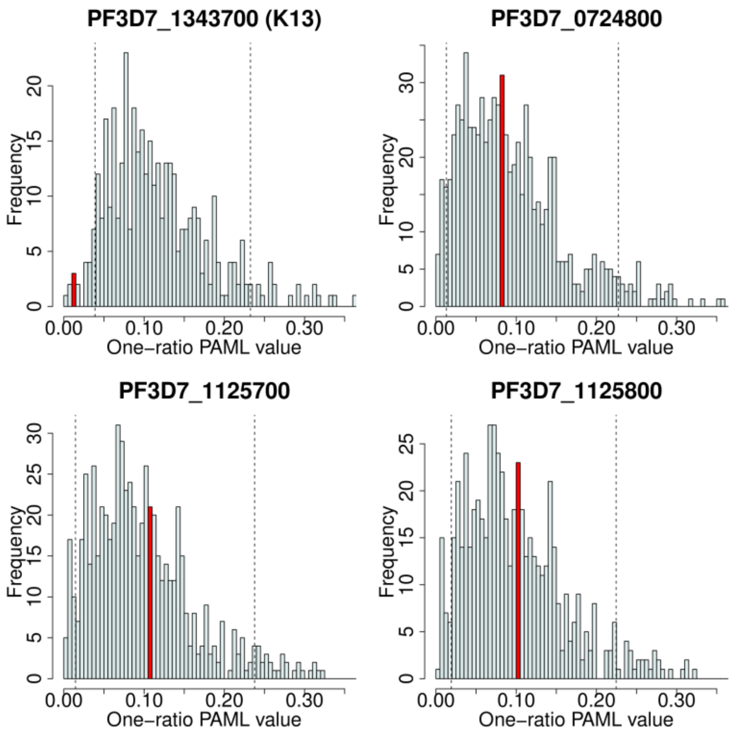

Figure S5. Conservation level of *k13* and four other KREP-containing protein-coding sequences compared to all *Plasmodium* genes.

The conservation level (d_N/d_S) was estimated for 3,256 orthologous genes among six *Plasmodium* species (*P. falciparum*, *P. berghei*, *P. chabaudi*, *P. vivax*, *P. yoelii* and *P. knowlesi)* under the one-ratio PAML model. Histograms show the distribution of one-ratio PAML values for protein-coding genes whose length is comprised in an interval of ± 100 amino acids centered on the length of each KREP-containing protein (sized-rank) which corrects for a correlation between protein length and one-ratio PAML value. PlasmoDB accession numbers of the five KREP-containing proteins investigated are provided above each histogram. Red bars indicate the position of each of these KREP-containing proteins in the d_N/d_S distribution, with vertical dashed lines showing the five percent cutoff of the most and less conserved protein-coding genes.

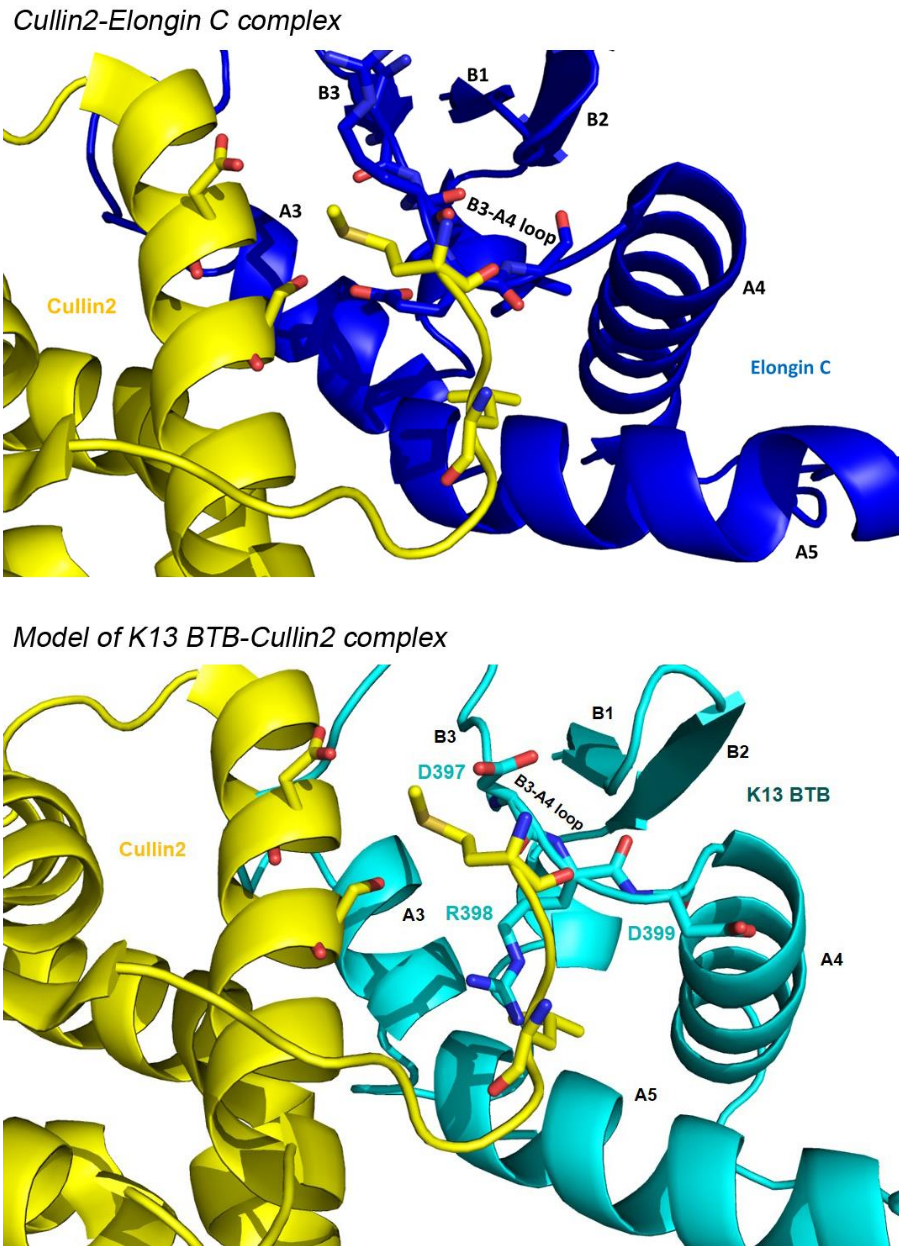

Figure S6. Model of the K13 BTB-Cullin2 interaction using the X-ray structure of the Elongin C-Cullin2 complex as reference.

K13 BTB, Elongin C and Cullin2 are shown in cyan, blue and yellow, respectively. The B3-A4 loop in Elongin C contains amino acids that are reported to make contacts with Cullin2 (*upper structure*). The amino acid sites forming the B3-A4 loop of K13 BTB (positions 397-399 using the PfK13 sequence numbering) are strictly conserved during *Apicomplexa* evolution. The structural alignment of K13 BTB with Elongin C showed that amino acid sites 397 and 398 are oriented towards the Cullin protein with contacts distance ≤ 3.0 Å (*lower structure*). The structures were visualized with PyMOL (Schrödinger, LLC).

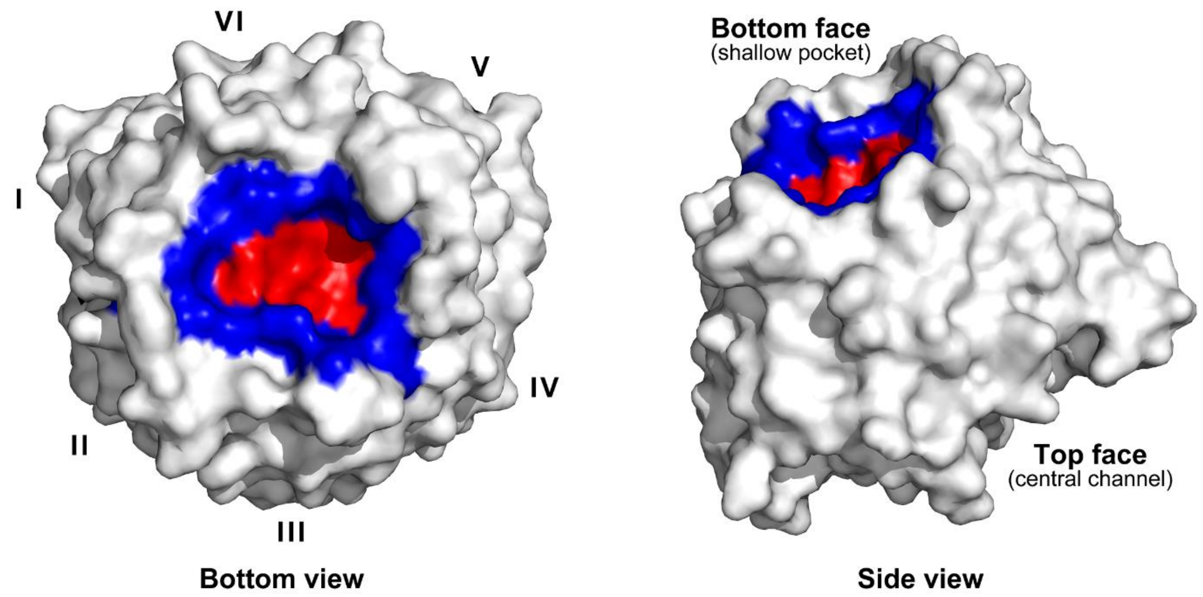

Figure S7. Structural delineation of the shallow pocket in the K13 KREP domain.
No conventional definition exists for the shallow pocket delineation in KREP folds. Consequently, we defined it as the amino acids forming the surface plan of the pocket plus those protruding out of the plan. We used using the PfK13 BTB-KREP structure solved by X-ray diffraction at a resolution of 1.81 Å (PDB code 4YY8, chain A). The surface plan of the K13 shallow pocket included the positions 482, 530, 576, 577, 623, 624 and 672 (n = 7), shown in red. The amino acid positions protruding out of the plan included the positions 451, 456, 498, 529, 546, 551, 593, 597, 640, 671, 688 and 717 (n = 12), shown in blue. The structure is shown as surface and visualized with PyMOL (Schrödinger, LLC).

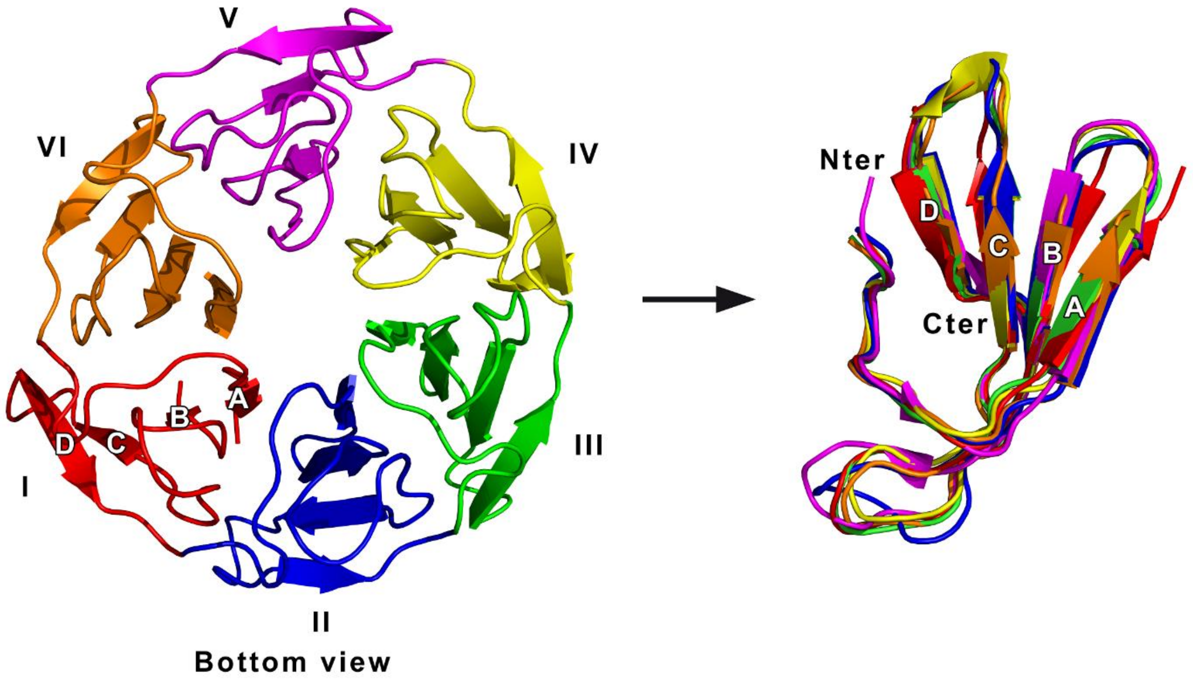

Figure S8. Structure-based alignment of the six blade repeats of PfK13 KREP.

We used using the PfK13 BTB-KREP structure solved by X-ray diffraction at a resolution of 1.81 Å (PDB code 4YY8, chain A). The PfK13 KREP structure is shown as cartoon. The six blades are colored differently: red, blade I; blue, blade II; green, blade III; yellow, blade IV; magenta, blade V; orange, blade VI. The four strands within each blade are labelled A to D, from the innermost to the outermost. The KREP structure was divided into six PDB files, each one containing the atomic coordinates of one blade. Then, the six blade structures were aligned by minimizing the root-mean-square deviation (RMSD) of atomic positions using the *align* function in PyMOL (Schrödinger, LLC) (*right*). This structure-based alignment of the K13 KREP blades was then used to produce the amino acid sequence alignment of the six blades shown in Figure 4a. The blade length ranged from 46 (blade VI) to 49 (blade V) amino acids, the pairwise blade sequence conservation level ranged from 15.2% (blades III/VI) to 33.3% (blades IV/V), and the pairwise blade structure comparison indicated a RMSD ranging from 0.394 Å^2 (blades I/III) to 0.669 Å^2 (blades I/IV).

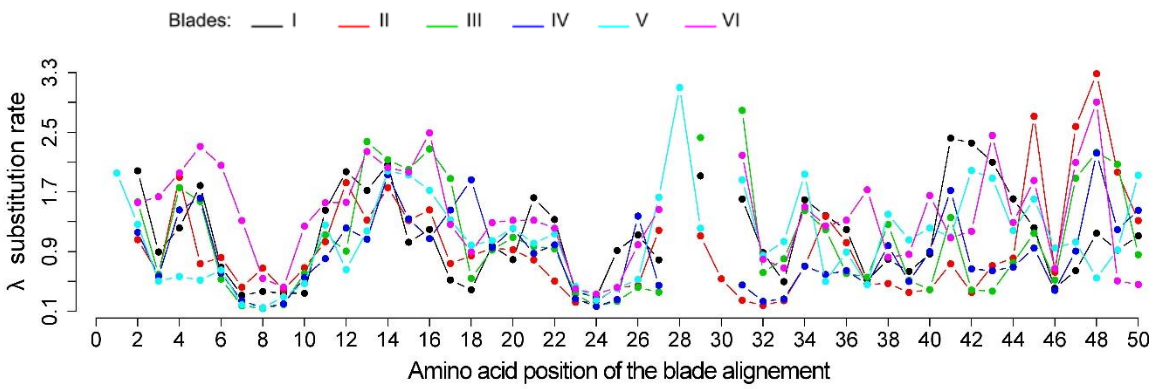

Figure S9. Conservation level of the amino acid sites for each blade along the structure-based amino acid alignment of the six blades.

Conservation level of amino acids (λ substitution rate) was estimated with the FuncPatch server. Each blade is associated with a specific color. Because the structure-based amino acid alignment of the six blades contained some gaps, there are missing values.

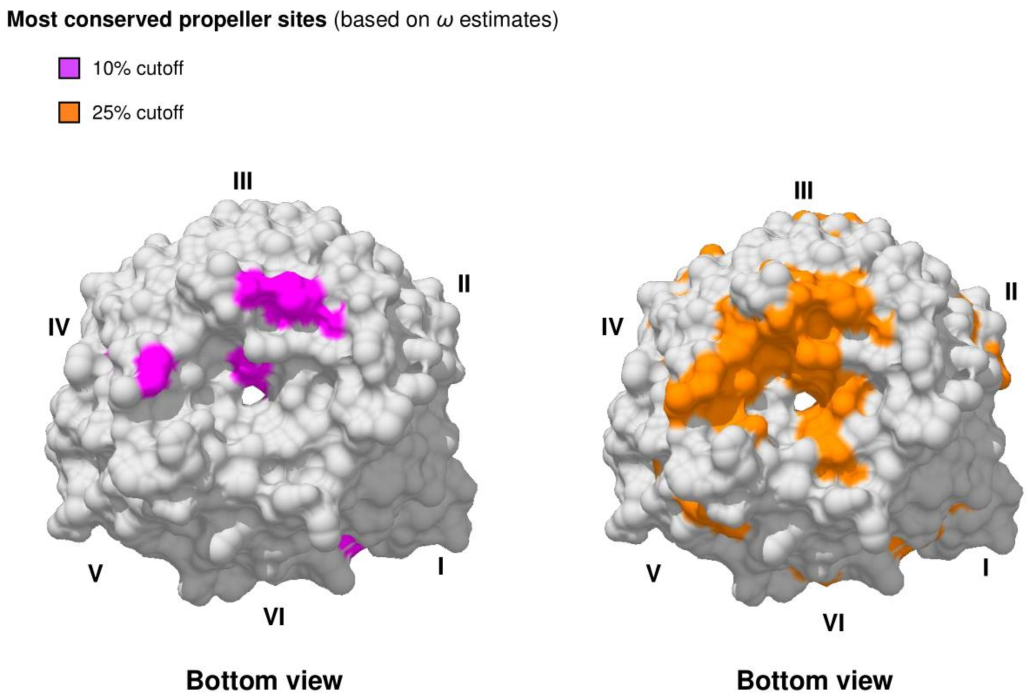

Protein	Conservation level	Shallow pocket positions	Remaining positions	*p value*
K13	High$^{10\%}$	1	27	0.704
	Others	18	238	
	High$^{25\%}$	10	61	6.9×10^{-3}
	Others	9	204	

Figure S10. Conservation level of the amino acid sites forming the shallow pocket of K13 KREP, estimated using PAML data.

Conservation level of amino acid sites (ω substitution rate) was estimated with the PAML program which does not take into account the protein tertiary structure. The 10% (*left structure*) and 25% (*right structure*) most conserved sites are mapped on the three-dimensional structure of PfK13 KREP in magenta and orange, respectively. Only one position of the shallow pocket belonged to the 10% of the most conserved positions (position 530 using the PfK13 sequence numbering). This result is consistent with initial reports by FuncPatch' authors. Without information provided by the tertiary structure, the most conserved positions of K13 KREP inferred by PAML corresponded to the signature amino acids of KREP proteins: the conserved arginine/lysine (R/K) in DA loops, the diglycine doublet (GG) after B strands, and the tryptophan (W) in C strands. However, when we extended the cutoff to the 25% most conserved sites, the shallow pocket was statistically enriched in conserved positions (10/19 positions: 482, 529, 546, 551, 576, 593, 597, 671, 672 and 688). Contingency tables were subjected to chi-squared or Fisher's exact tests.

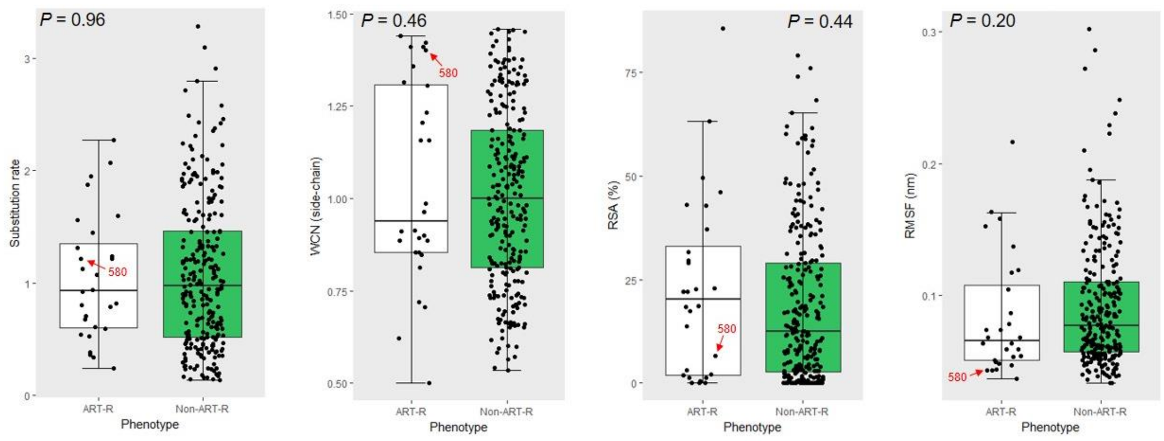

Figure S11. Absence of significant difference in evolutionary- and structure-based parameters between K13 KREP positions associated or not with an ART-R mutation.

Box plots showing the distribution of, from *left* to *right* respectively: the site-specific substitution rates λ inferred from FuncPatch analysis, the relative solvent accessibility (RSA) values, the side-chain weighted contact number (WCN_{sc}) values and the root-mean-square fluctuation (RMSF) values. λ, RSA, WCN_{sc}, and RMSF values were calculated using the PfK13 BTB-KREP structure solved by X-ray diffraction at a resolution of 1.81 Å (PDB code 4YY8, chain A). K13 KREP positions were categorized as either associated with a reported ART-R mutation (*ART-R group*, white) or not (*non-ART-R group*, green). All values are provided in the Supplementary Dataset S2. The position 580 – associated with the predominant *pfk13* C580Y allele in SEA – is indicated by the red arrow. Box boundaries represent the first and third quartiles and the length of whiskers correspond to 1.5 times the interquartile range. The difference between groups was evaluated by non-parametric Mann-Whitney *U* test.

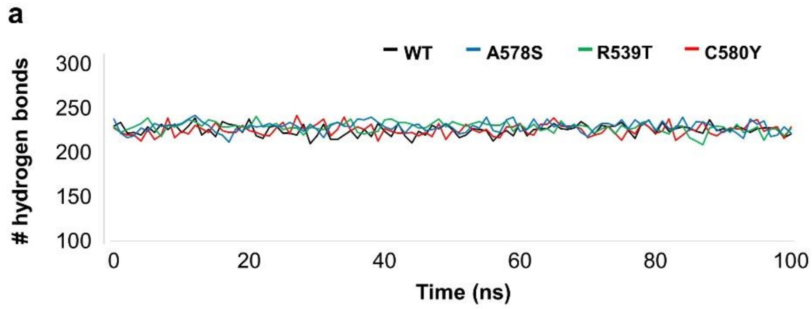

	Mean # H-bonds
WT	225 ± 6
A578S	229 ± 6
R539T	229 ± 6
C580Y	225 ± 6

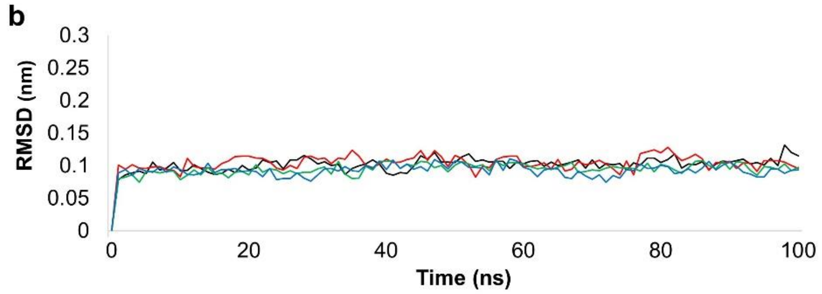

	Mean RMSD (nm)
WT	0.103 ± 0.008
A578S	0.093 ± 0.008
R539T	0.096 ± 0.007
C580Y	0.105 ± 0.009

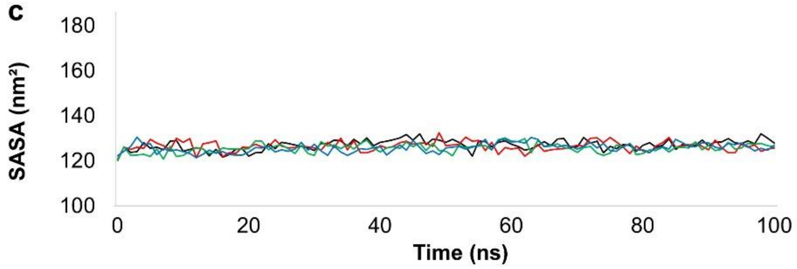

	Mean SASA (nm²)
WT	127.1 ± 2.3
A578S	125.8 ± 2.0
R539T	125.7 ± 2.1
C580Y	126.5 ± 2.2

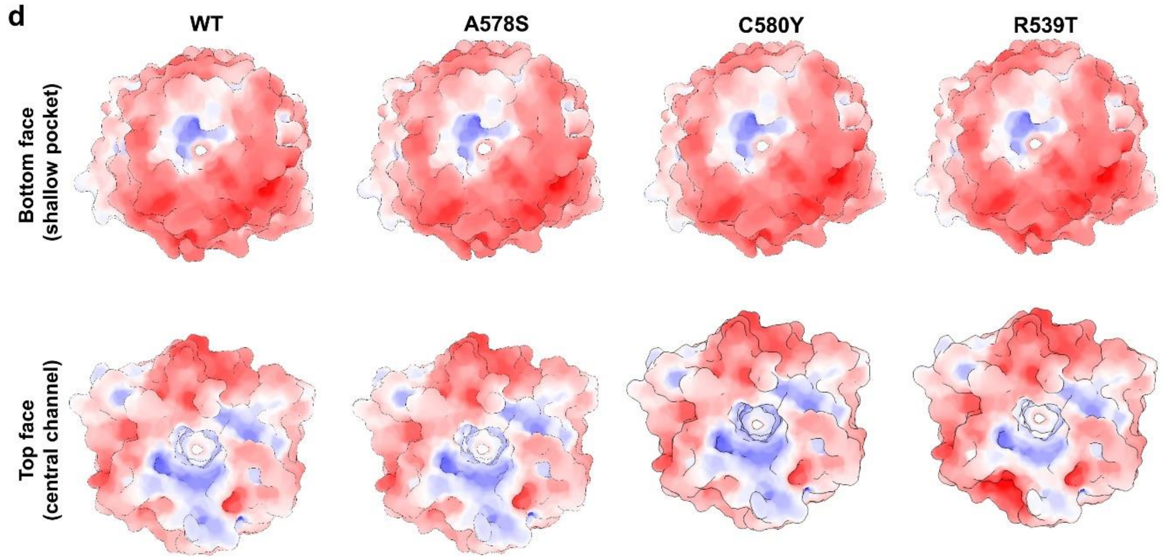

Figure S12. Global impacts of the A578S, C580Y and R539T mutations on the PfK13 KREP structures, as assessed by molecular dynamics simulations.

Molecular dynamics simulations were carried out on the KREP structure using GROMACS during 100 ns at a temperature of 300 K in an all-atom system. The first five nanoseconds (ns) correspond to the equilibration phase. Results were saved at each ns considering all-atom KREP structures. C580Y and R539T are two common mutations conferring ART-R in Southeast Asian parasites. A578S

mutation is commonly found in African parasites but does not confer ART-R. **(a)** Root-mean-square deviation (RMSD) values of wild type and mutant PfK13 KREP structures. Wild type and mutant structures were very stable and followed similar trajectories, with a mean RMSD close to 0.1 nm. **(b)** Evolution of hydrogen bond number during trajectories. A hydrogen bond was counted when the donor-acceptor distance is ≤ 3.5 Å and donor-hydrogen-acceptor angle ≤ 30°. Wild type and mutant structures showed similar average number of hydrogen bonds. **(c)** Solvent accessible surface area (SASA) values of wild type and mutant PfK13 KREP structures. Wild type and mutant structures showed similar SASA, suggesting no drastic changes in the KREP fold. **(d)** Electrostatic surface potential of wild type and mutant PfK13 KREP structures, estimated with the APBS method. Electrostatic potential values are in units of *kT*/*e* at 298 K, on a scale of -8 *kT/e* (red) to +8 *kT/e* (blue). White color indicates a neutral potential. The electrostatic potential is shown for the bottom face (shallow pocket) and for the top face (central channel). Wild type and mutant structures showed similar electrostatic surface potentials at the end of the simulations.

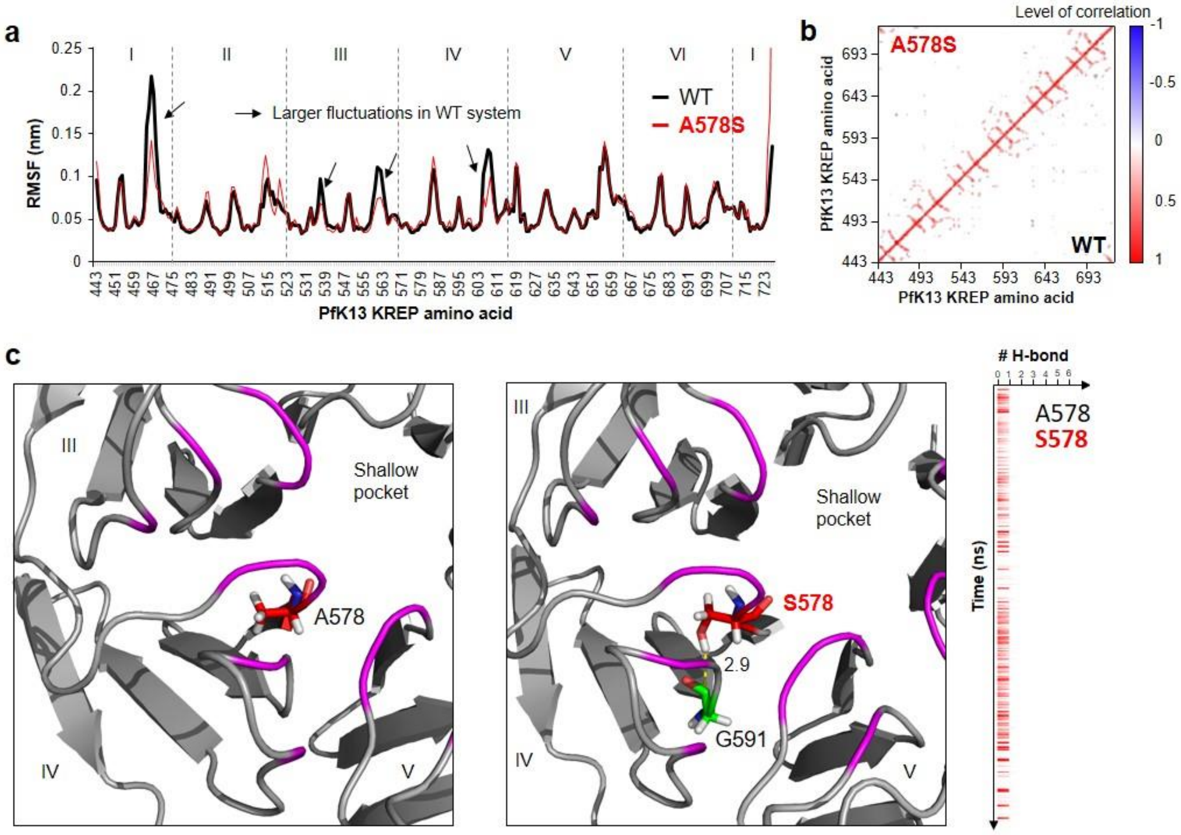

Figure S13. Local impacts of the A578S mutation on the PfK13 KREP structure, as assessed by molecular dynamics simulations. Molecular dynamics simulations on wild type and A578S mutant PfK13 KREP structure were carried out using GROMACS during 100 ns at a temperature of 300 K in an all-atom system. The first five nanoseconds (ns) correspond to the equilibration phase. **(a)** Root-mean-square fluctuation (RMSF) values of wild type and A578S mutant PfK13 KREP structures. RMSF per position was calculating on Cα atoms (excluding the first five ns, corresponding to the equilibration phase). **(b)** Dynamical cross-correlation maps (DCCMs) of wild type and A578S mutant PfK13 KREP structures. Wild type and mutant systems are shown in *bottom right* and *top left*, respectively. Positive or negative correlation for a pair of residue implies that the two residues move in the same (red) or opposition (blue) directions, respectively. No major difference in correlated and anti-correlated motions of KREP residues was found between the A578S ART-S and the wild type systems. Maps were generated using Bio3D in R. **(c)** Local impacts of A578S ART-S mutation on PfK13 KREP structure. Blade number (I to VI) and location of the shallow pocket are indicated. We observed the presence of one recurrent hydrogen bond (G591, blade IV), which was not found in the wild type system.

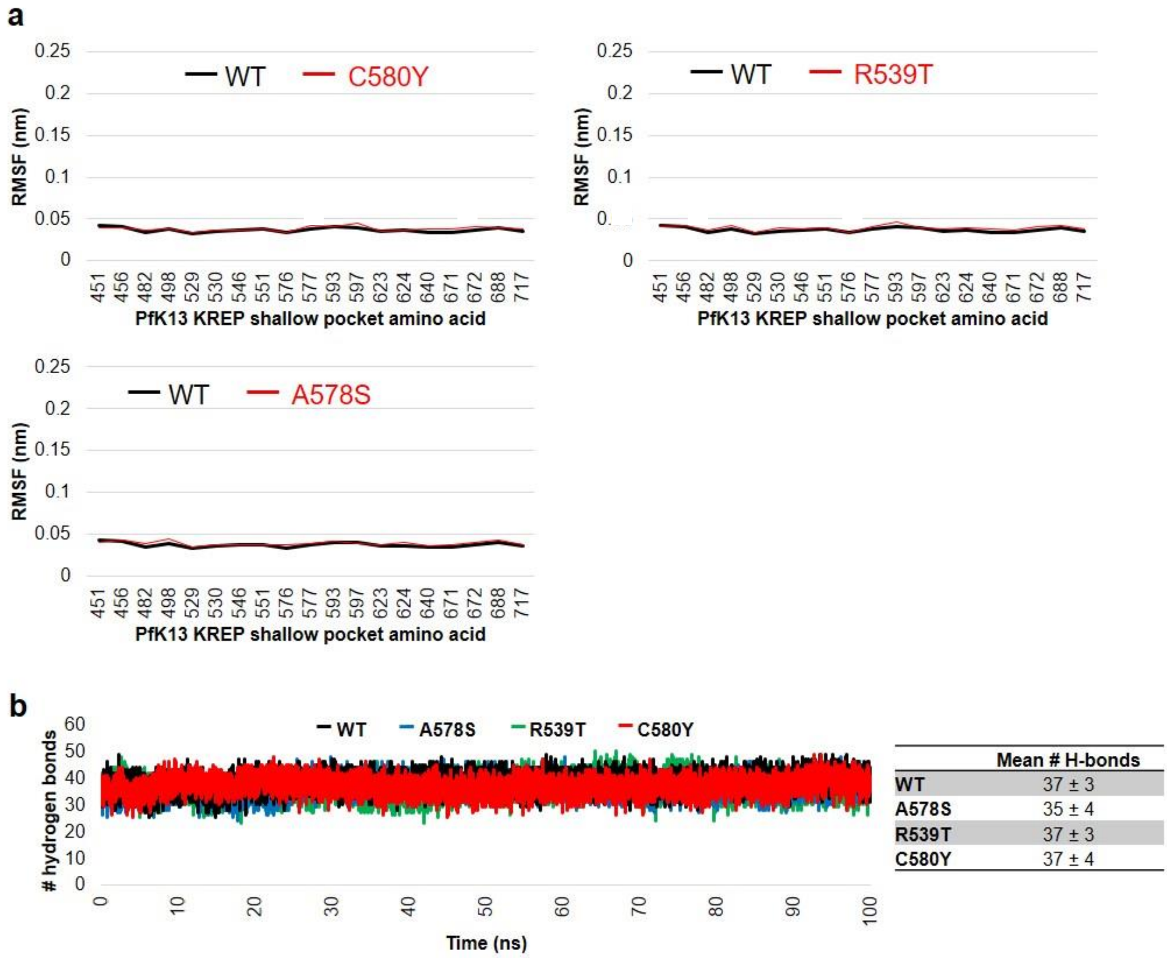

	Mean # H-bonds
WT	37 ± 3
A578S	35 ± 4
R539T	37 ± 3
C580Y	37 ± 4

Figure S14. Lack of difference in the number of H-bonds and amino acid fluctuations (RMSFs) between WT and mutant systems for amino acids forming the shallow pocket. Molecular dynamics simulations were carried out on the KREP structure using GROMACS during 100 ns at a temperature of 300 K in an all-atom system. The first ns (0 to 5 ns) correspond to an equilibration phase. **(a)** WT-mutant comparison of the root-mean-square fluctuations (RMSFs) associated to residues forming the shallow pocket. RMSF per position was calculating on cα atoms (excluding the first five ns, corresponding to the equilibration phase). WT and mutants are shown in black and red colors. **(b)** Evolution of hydrogen bond number for amino acids forming the shallow pocket during trajectories. A hydrogen bond was counted when the donor-acceptor distance is ≤ 3.5 Å and donor-hydrogen-acceptor angle ≤ 30°. Wild type and mutant structures showed similar average number of hydrogen bonds. No crucial hydrogen bonds were lost in any of the simulations.
