# Supplementary material for: Comparative structural and evolutionary analyses predict functional sites in the artemisinin resistance malaria protein K13
Source: Sci Rep. 2019 Jul 23;9:10675. doi: 10.1038/s41598-019-47034-6 (PMC6650413; doi:10.1038/s41598-019-47034-6)
Supplement: Supplementary file 2 — Supplementary Tables [file 41598_2019_47034_MOESM2_ESM.pdf]

Supplementary Tables

This supplemental file has been provided by the authors to give readers additional information about their work.

Supplement to: **Comparative structural and evolutionary analyses predict functional sites in the artemisinin resistance malaria protein K13**

By Romain Coppée, Daniel C. Jeffares, Maria A. Miteva, Audrey Sabbagh, Jérôme Clain

Table S1 .... **2**

Table S2 .... **4**

Table S3 .... **5**

Table S4 .... **6**

Table S5 .... **7**

Table S6 .... **8**

Note : Some tables are on multiple pages.

Table S1. List of K13 orthologous sequences used in this study.

Accession Number [a]	Species	Abbreviation [b]	Description	Source	Length [c]	Hosts [d]
PF3D7_1343700	*Plasmodium falciparum*	Pfal	Kelch protein kelch-13	PlasmoDB	726	Humans
XP_012764805.1	*Plasmodium reichenowi*	Prei	Kelch protein, putative	NCBI	724	Monkeys
XP_018640212.1	*Plasmodium gaboni*	Pgab	Putative kelch protein K13	NCBI	725	Monkeys
XP_001614215.1	*Plasmodium vivax*	Pviv	Kelch domain-containing protein	NCBI	712	Humans / Monkeys
XP_008815744.1	*Plasmodium inui*	Pinu	Hypothetical protein C922_01923	NCBI	712	Monkeys
XP_002259918.1	*Plasmodium knowlesi*	Pkno	Kelch-motif containing protein	NCBI	712	Humans / Monkeys
XP_012337473.1	*Plasmodium fragile*	Pfra	Hypothetical protein AK88_04440	NCBI	712	Monkeys
PCYB_122000	*Plasmodium cynomolgi*	Pcyn	Kelch domain-containing protein	PlasmoDB	713	Monkeys
XP_008622491.1	*Plasmodium vinckei vinckei*	Pvin	Hypothetical protein YYE_00585	NCBI	738	Rodents
EUD71747.1	*Plasmodium vinckei petteri*	Ppet	Hypothetical protein YYG_03164	NCBI	738	Rodents
CDR16038.1	*Plasmodium chabaudi chabaudi*	Pcha	Kelch protein kelch-13, putative	NCBI	738	Rodents
SCM11204.1	*Plasmodium chabaudi adami*	Pada	Kelch protein K13, putative	NCBI	738	Rodents
XP_730901.1	*Plasmodium yoelii*	Pyoe	Hypothetical protein	NCBI	738	Rodents
CDS51002.1	*Plasmodium berghei*	Pber	Kelch protein kelch-13, putative	NCBI	738	Rodents
PCOAH_00041530	*Plasmodium coatneyi*	Pcoa	Uncharacterized protein	PlasmoDB	712	Monkeys
PGAL8A_00254800	*Plasmodium gallinaceum*	Pgal	Kelch protein K13, putative	PlasmoDB	706	Birds
PRELSG_1254200	*Plasmodium relictum*	Prel	Kelch protein K13, putative	PlasmoDB	704	Birds
GAW82138.1	*Plasmodium gonderi*	Pgon	Kelch protein K13	NCBI	712	Monkeys
PmUG01_12021200	*Plasmodium malariae*	Pmal	Kelch protein K13, putative	PlasmoDB	714	Humans
ANG83523.1	*Plasmodium ovale wallikeri*	Pwal	Kelch protein K13, partial	NCBI	687	Humans
PocGH01_12019400	*Plasmodium ovale curtisi*	Pcur	Kelch protein K13, putative	PlasmoDB	725	Humans
ESS31969.1	*Toxoplasma gondii*	Tgon	Kelch repeat and K+ channel tetramerisation domain containing protein	NCBI	818	—
XP_008882882.1	*Hammondia hammondi*	Hham	Kelch repeat and K+ channel tetramerisation domain containing protein	NCBI	820	—
XP_003882792.1	*Neospora caninum*	Ncan	Kelch motif domain-containing protein	NCBI	804	—
XP_013247290.1	*Eimeria acervulina*	Eace	Kelch motif domain-containing protein	NCBI	654	—
EfaB_MINUS_11414	*Eimeria falciformis*	Efal	Unspecified product	ToxoDB	662	—
CDJ54128.1<br>Ebh_scaff_2461<br>Ebh_scaff_22	*Eimeria brunetti*	Ebru	Kelch motif domain-containing protein, putative	NCBI<br>GeneDB	506	—
XP_013334533.1<br>Ema_scaff_872	*Eimeria maxima*	Emax	Kelch motif domain-containing protein, putative	NCBI<br>GeneDB	509	—

Accession Number [a]	Species	Abbreviation [b]	Description [c]	Source	Length [d]	Hosts [e]
XP_012648586.1	*Babesia microti*	Bmic	Uncharacterized protein	NCBI	557	—
XP_766008.1	*Theileria parva*	Tpar	Hypothetical protein	NCBI	576	—
XP_004829715.1	*Theileria equi*	Tequ	Kelch repeat domain containing protein	NCBI	556	—
XP_009689263.1	*Theileria orientalis*	Tori	Uncharacterized protein TOT_010000427	NCBI	603	—
XP_001609009.1	*Babesia bovis*	Bbov	Kelch repeat and K+ channel tetramerisation domain containing protein	NCBI	569	—
tann.chr01	*Theileria annulata*	Tann	Protein with kelch domain	GeneDB	576	—
XP_012767816.1	*Babesia bigemina*	Bbig	Kelch repeat and K+ channel tetramerisation domain containing protein, putative	NCBI	634	—
XP_002141433.1	*Cryptosporidium muris*	Cmur	Kelch motif family protein	NCBI	561	—
XP_667484.1	*Cryptosporidium hominis*	Chom	Hypothetical protein	NCBI	579	—
CmeUKMEL1_04065	*Cryptosporidium meleagridis*	Cmel	Kelch motif family protein	CryptoDB	580	
cubi_02935	*Cryptosporidium ubiquitum*	Cubi	KELCH domain-containing protein	CryptoDB	580	—
Cbai_ctg7180000000685	*Cryptosporidium baileyi*	Cbai	—	CryptoDB	566	—
cand_030750	*Cryptosporidium andersoni*	Cand	Kelch motif family protein	CryptoDB	561	—
XP_628432.1	*Cryptosporadium parvum*	Cpar	POZ+kelch domain protein with kelch repeats at the C-terminus	NCBI	580	—
SN3_01300125	*Sarcocystis neurona*	Sneu	Kelch protein	ToxoDB	707	—

[a] Identifier of the protein sequence retrieved from sequence databases.

[b] Four-letter species code used in Supplementary Figure S1.

[c] Number of amino acids.

[d] Vertebrate host(s) are only provided for *Plasmodium* species.

Table S2. Results of PAML analyses for the *k13* gene.

Model	*l*	Parameters	κ	Positively selected codons
FR (free-ratio)	-29,511.0	ω can vary between branches	1.19	NA
M0 (one-ratio)	-29,739.4	ω = 0.022	1.39	NA
M1a (neutral)	-29,727.8	ω_0 = 0.021, p_0 = 0.994<br>ω_1 = 1.000, p_1 = 0.006	1.41	NA
M2a (selection)	-29,727.8	ω_0 = 0.021, p_0 = 0.994<br>ω_1 = 1.000, p_1 = 0.002<br>ω_2 = 1.000, p_2 = 0.004	1.41	None detected
M3 (discrete)<br>(*k* = 5)	-29,216.3	ω_0 = 0.0002, p_0 = 0.105<br>ω_1 = 0.0065, p_1 = 0.245<br>ω_2 = 0.0202, p_2 = 0.420<br>ω_3 = 0.0491, p_3 = 0.210<br>ω_4 = 0.1100, p_4 = 0.020	1.43	None detected
M7 (beta)	-29,225.2	β(0.98, 40.98)<br>$\omega \leq$ 1.00, p = 1.000	1.43	NA
M8 (beta&ω)	-29,225.2	β(0.98, 40.98)<br>$\omega \leq$ 1.00, p_0 = 0.999	1.43	None detected

ω, d_N/d_S ratio; p_n, proportion of codon sites in the site class ω_n; *l*, log-likelihood value; κ, transition/transversion rate ratio; $\beta(p, q)$, shape parameters for the β-distribution of ω. Candidate codon sites for positive selection are identified using the Bayes empirical Bayes (BEB) inference for models M2a and M8, and the Naïve empirical Bayes (NEB) inference for model M3.

Table S3. Distribution of the conserved KREP shallow pocket positions for K13 and some BTB-Kelch proteins.

Protein	Conservation level [a]	Shallow pocket positions [b]	Remaining positions	*p value*
$K13_{KREP}$	High	9	20	1.60×10^{-5}
	Others	10	245	
$KEAP1_{KREP}$	High	12	17	1.44×10^{-7}
	Others	12	244	
$KLHL2_{KREP}$	High	13	16	1.26×10^{-10}
	Others	6	251	
$KLHL3_{KREP}$	High	12	17	1.36×10^{-9}
	Others	6	251	
$KLHL12_{KREP}$	High	14	15	2.94×10^{-12}
	Others	5	255	

[a] The KREP positions were categorized into two groups: the *high* conservation level group contained the 10% most conserved positions according to the λ substitution rates, the *others* group included the KREP positions with lower levels of conservation. [b] The shallow pocket of K13 KREP was defined as the positions forming the surface plan of the pocket and protruding out of the plan. A view and delineation of the PfK13 KREP shallow pocket is provided in Supplementary Figure S7. The same structural delineation of the KREP shallow pocket was applied to the four other BTB-Kelch KREP domains. Contingency tables were subjected to chi-squared test.

Table S4. Conservation level of the protein sites associated with artemisinin resistance or artemisinin sensitive mutations.

AA [a] position	Ref. AA [a] (*P. fal*)	PfK13 mutant [b]	Amino acid variation across *Apicomplexa* species: *Plasmodium* (n = 21)	Amino acid variation across *Apicomplexa* species: Non-*Plasmodium* (n = 22)	λ-based rank n, (%) [c]
**446**	**F**	**F446I**	**F (21)**	**F (9); Y (7); L (6)**	**110 (38.73)**
449	G	G449A	G (21)	G (22)	32 (11.27)
452	D	D452E	D (21)	N (14); S (4); D (4)	164 (57.75)
**458**	**N**	**N458Y**	**N (20); S (1)**	**N (6); D (6); R (5); K (2); S (1); G (1); P (1)**	**222 (78.17)**
469	C	C469Y	C (21)	C (10); G (4) ; V (3); R (2); K (1); Q (1); N (1)	185 (65.14)
**476**	**M**	**M476I**	**M (21)**	**M (21) ; L (1)**	**81 (28.52)**
479	K	K479I	K (21)	E (13); P (4); A (3) ; S (2)	117 (41.20)
481	A	A481V	A (21)	A (13); M (7); I (1); T (1)	96 (33.80)
**493**	**Y**	**Y493H**	**Y (21)**	**Y (10); A (5); F (4); C (3)**	**136 (47.89)**
515	R	R515K	R (21)	R (12); I (4); L (3); T (3)	103 (36.27)
522	S	S522C	S (21)	A (13); P (5); K (2); S (1); Q (1)	194 (68.31)
527	P	P527H	P (21)	P (13); G (5); A (4)	71 (25.00)
537	N	N537I; N537D	N (21)	D (10); G (5); E (2); N (2); S (1); A (1); R (1)	271 (95.42)
538	G	G538V	G (21)	G (10); N (3); D (3); E (2); S (2); A (1); K (1)	242 (85.21)
**539**	**R**	**R539T**	**R (21)**	**R (22)**	**74 (26.06)**
**543**	**I**	**I543T**	**I (21)**	**I (10); V (10); A (2)**	**137 (48.24)**
**553**	**P**	**P553L**	**P (21)**	**S (10); D (6); K (3); A (2); G (1)**	**209 (73.59)**
**561**	**R**	**R561H**	**R (21)**	**R (22)**	**36 (12.68)**
568	V	V568G	V (13); I (8)	V (9); L (4); S (3); A (2); G (2); M (1); C (1)	263 (92.61)
574	P	P574L	P (21)	P (20); A (2)	85 (29.93)
575	R	R575K	R (21)	R (22)	15 (5.28)
#578	A	A578S	A (12); S (8); C (1)	A (20); P (2)	76 (26.76)
579	M	M579I	M (21)	M (15); S (6); C (1)	114 (40.14)
**580**	**C**	**C580Y**	**C (19); S (2)**	**C (14); V (4); L (2); I (1); A (1)**	**183 (64.44)**
584	D	D584V	D (20); E (1)	D (9); N (7); G (4); E (2)	158 (55.63)
667	P	P667T	P (21)	P (12); E (3); A (2); C (1); S (1); I (1); D (1); N (1)	252 (88.73)
673	F	F673I	F (21)	C (11); A (5); S (3); T (2); G (1)	186 (65.49)
675	A	A675V	A (17); S (4)	A (8); I (6); M (4); L (2); V (2)	219 (77.11)
719	H	H719N	H (20); ? (1)	H (22)	28 (9.86)

Note – [a] AA: amino acid position. Ref. AA indicates the wild type residue in PfK13. [b] Validated and candidate PfK13 artemisinin resistance mutations (according to WHO) are shown in bold and normal letters, respectively; A578S (indicated with the # symbol) does not confer resistance to artemisinin. [c] The rank attributed to positions was based on the site-specific substitution rates λ (Supplementary Dataset S1) estimated for the 284 studied Kelch-repeat propeller (KREP) positions using FuncPatch. The lower the rank, the more conserved was the position.

Table S5. List of BTB-containing sequences used for the different BTB-containing protein families.

Accession Number [a]	Protein	Protein family [b]	Species	Length	Description [c]	Extended BTB [d]
Q8H1D3	NPY1	BTB-NPH3	*Arabidopsis thaliana*	571	BTB/POZ domain-containing protein NPY1	yes (C-ter only)
O80970	NPY2	BTB-NPH3	*Arabidopsis thaliana*	634	BTB/POZ domain-containing protein NPY2	yes (C-ter only)
Q9FN09	NPY3	BTB-NPH3	*Arabidopsis thaliana*	579	BTB/POZ domain-containing protein NPY3	yes (C-ter only)
O64814	NPY4	BTB-NPH3	*Arabidopsis thaliana*	481	BTB/POZ domain-containing protein NPY4	yes (C-ter only)
Q0WL52	NPY5	BTB-NPH3	*Arabidopsis thaliana*	580	BTB/POZ domain-containing protein NPY5	yes (C-ter only)
O15156	ZBTB7B	BTB-ZF	*Homo sapiens*	539	Zinc finger and BTB domain-containing protein 7B	yes (C-ter only)
Q96BR9	ZBTB8A	BTB-ZF	*Homo sapiens*	441	Zinc finger and BTB domain-containing protein 8A	yes (C-ter only)
Q05516	ZBTB16	BTB-ZF	*Homo sapiens*	673	Zinc finger and BTB domain-containing protein 16	yes (C-ter only)
Q9HC78	ZBTB20	BTB-ZF	*Homo sapiens*	741	Zinc finger and BTB domain-containing protein 20	yes (C-ter only)
O43298	ZBTB43	BTB-ZF	*Homo sapiens*	467	Zinc finger and BTB domain-containing protein 43	yes (C-ter only)
O95198	KLHL2	BTB-Kelch	*Homo sapiens*	593	Kelch-like protein 2	yes
Q53G59	KLHL12	BTB-Kelch	*Homo sapiens*	568	Kelch-like protein 12	yes
Q14145	KLHL19	BTB-Kelch	*Homo sapiens*	624	Kelch-like ECH-associated protein 1	yes
O60662	KLHL41	BTB-Kelch	*Homo sapiens*	606	Kelch-like protein 41	yes
O94844	RHOBTB1	RhoBTB	*Homo sapiens*	696	Rho-related BTB domain-containing protein 1	yes
Q9BYZ6	RHOBTB2	RhoBTB	*Homo sapiens*	727	Rho-related BTB domain-containing protein 2	yes
O94955	RHOBTB3	RhoBTB	*Homo sapiens*	611	Rho-related BTB domain-containing protein 3	yes
Q8L765	BPM1	MATH-BTB	*Arabidopsis thaliana*	407	BTB/POZ and MATH domain-containing protein 1	yes
Q9M8J9	BPM2	MATH-BTB	*Arabidopsis thaliana*	406	BTB/POZ and MATH domain-containing protein 2	yes
O22286	BPM3	MATH-BTB	*Arabidopsis thaliana*	408	BTB/POZ and MATH domain-containing protein 3	yes
Q9SRV1	BPM4	MATH-BTB	*Arabidopsis thaliana*	465	BTB/POZ and MATH domain-containing protein 4	yes
Q1EBV6	BPM5	MATH-BTB	*Arabidopsis thaliana*	410	BTB/POZ and MATH domain-containing protein 5	yes
Q8TBC3	SHKBP1	KCTD-like	*Homo sapiens*	707	SH3KBP1-binding protein 1	no
Q719H9	KCTD1	KCTD	*Homo sapiens*	257	BTB/POZ domain-containing protein KCTD1	no
Q8NC69	KCTD6	KCTD	*Homo sapiens*	237	BTB/POZ domain-containing protein KCTD6	no
Q8WZ19	KCTD13	KCTD	*Homo sapiens*	329	BTB/POZ domain-containing adapter for CUL3-mediated RhoA degradation protein 1	no
Q8N5Z5	KCTD17	KCTD	*Homo sapiens*	321	BTB/POZ domain-containing protein KCTD17	no
Q09470	KCNA1	KCNA	*Homo sapiens*	495	Potassium voltage-gated channel subfamily A member 1	no
P16389	KCNA2	KCNA	*Homo sapiens*	499	Potassium voltage-gated channel subfamily A member 2	no
P22001	KCNA3	KCNA	*Homo sapiens*	575	Potassium voltage-gated channel subfamily A member 3	no
P22459	KCNA4	KCNA	*Homo sapiens*	653	Potassium voltage-gated channel subfamily A member 4	no
P22460	KCNA5	KCNA	*Homo sapiens*	613	Potassium voltage-gated channel subfamily A member 5	no
P63208	SKP1	—	*Homo sapiens*	163	S-phase kinase-associated protein 1	yes (N-ter only)
Q15369	Elongin C	—	*Homo sapiens*	112	Elongin-C	no

[a] Identifier of the protein sequence retrieved from the UniProtKB database.

[b] BTB-containing proteins are classified into different families or subfamilies on the basis of the protein domain composition.

[c] Functional annotation of the protein, provided by UniprotKB database.

[d] BTB domains from the BTB-ZF, BTB-NPH3, BTB-Kelch, RhoBTB and MATH-BTB proteins contain additional N-terminal and/or C-terminal structural elements.

Table S6. Full list of species and GenBank entries used for SHKBP1, KCTD17, KEAP1, KLHL2, KLHL3 and KLHL12 proteins.

	GenBank accession numbers					
**Species**	**SHKBP1 (n = 124)**	**KCTD17 (n = 139)**	**KEAP1 (n = 135)**	**KLHL2 (n = 162)**	**KLHL3 (n = 158)**	**KLHL12 (n = 129)**
Acanthaster planci	—	—	—	XP_022079940.1	—	—
Acanthisitta chloris	—	XP_009082248.1	—	—	—	—
Acanthochromis polyacanthus	—	—	XP_022046307.1	XP_022074174.1	XP_022075435.1	XP_022043757.1
Acinonyx jubatus	—	—	XP_014933811.1	—	XP_014919947.1	—
Acropora digitifera	—	—	—	XP_015764133.1	—	—
Ailuropoda melanoleuca	—	—	XP_002921417.1	XP_011233125.2	XP_011227565.2	XP_002925475.1
Alligator mississippiensis	—	XP_019345267.1	—	XP_019336971.1	KYO20331.1	—
Alligator sinensis	—	XP_014376482.1	XP_006023504.1	XP_014374102.1	XP_014376891.1	XP_006038484.1
Amazona aestiva	—	KQK76492.1	—	—	—	—
Amphiprion ocellaris	XP_023125107.1	—	XP_023128544.1	XP_023135194.1	XP_023143999.1	XP_023131332.1
Anas platyrhynchos	—	EOB03950.1	—	—	XP_005018740.1	—
Anolis carolinensis	—	XP_008108870.1	XP_003216447.1	XP_008116333.1	XP_008103098.1	XP_003220513.1
Anser cygnoides domesticus	—	XP_013036383.1	—	XP_013030701.1	XP_013031336.1	—
Antrostomus carolinensis	—	XP_010160965.1	—	—	KFZ58705.1	—
Aotus nancymaae	—	XP_012331822.1	XP_012291394.1	XP_021530947.1	XP_012290872.1	—
Aplysia californica	—	—	—	XP_005099881.1	—	—
Aptenodytes forsteri	—	XP_009288225.2	—	XP_009270590.1	KFM12065.1	XP_009280168.1
Apteryx australis mantelli	—	XP_013802726.1	—	XP_013797640.1	—	—
Aquila chrysaetos canadensis	—	XP_011592211.1	—	XP_011572555.1	XP_011569540.1	—
Astyanax mexicanus	XP_022535160.1	—	XP_007231370.1	XP_022537500.1	XP_007246470.2	XP_022526735.1
Austrofundulus limnaeus	XP_013889136.1	—	XP_013860381.1	XP_013875667.1	—	XP_013871669.1
Balaenoptera acutorostrata scammoni	XP_007166868.1	XP_007165722.1	—	XP_007176776.1	—	—
Balearica regulorum gibbericeps	—	KFO11200.1	—	XP_010297540.1	—	—
Bison bison bison	XP_010847541.1	XP_010858924.1	XP_010826664.1	—	XP_010842774.1	—
Boleophthalmus pectinirostris	XP_020794892.1	—	XP_020794190.1	XP_020788618.1	XP_020779108.1	XP_020785201.1
Bos indicus	XP_019833810.1	XP_019816894.1	—	—	XP_019820522.1	—
Bos mutus	ELR60093.1	XP_014333583.1	ELR51175.1	XP_005898408.1	—	—
Bos taurus	NP_001076076.1	—	NP_001094612.1	XP_005217484.1	XP_612749.5	—
Branchiostoma belcheri	—	—	—	XP_019635414.1	—	—
Bubalus bubalis	XP_006050123.1	XP_006057052.1	XP_006068412.1	XP_006073262.1	—	XP_006078512.1
Buceros rhinoceros silvestris	—	XP_010143072.1	—	—	—	—
Calidris pugnax	—	XP_014819558.1	—	—	—	—
Callithrix jacchus	XP_008986284.1	XP_017819005.1	—	XP_002745302.1	—	XP_008983687.1
Callorhinchus milii	—	—	XP_007907175.1	XP_007904738.1	—	XP_007899790.1
Calypte anna	—	XP_008494604.1	—	XP_008492043.1	KFO97435.1	—
Camelus bactrianus	XP_010945566.1	XP_010958659.1	—	—	—	XP_010954536.1
Camelus dromedarius	—	XP_010993082.1	—	—	—	—

Species	SHKBP1 (n = 124)	KCTD17 (n = 139)	KEAP1 (n = 135)	KLHL2 (n = 162)	KLHL3 (n = 158)	KLHL12 (n = 129)
Camelus ferus	XP_006177980.1	—	XP_006179430.1	XP_006190874.1	XP_006180366.1	XP_006173510.2
Canis lupus familiaris	XP_533665.2	—	XP_533917.2	—	XP_005626632.1	XP_537119.2
Capra hircus	XP_017917921.1	XP_017903788.1	—	—	—	XP_013825912.1
Cariama cristata	—	XP_009708538.1	—	XP_009707350.1	XP_009707735.1	—
Carlito syrichta	—	—	XP_008049111.2	XP_021568132.1	—	—
Castor canadensis	XP_020022544.1	XP_020008051.1	XP_020029886.1	—	—	—
Cavia porcellus	XP_003462052.1	—	—	XP_003479693.2	XP_003473223.2	XP_003474875.1
Cebus capucinus imitator	XP_017358686.1	XP_017364405.1	—	—	XP_017356455.1	—
Ceratotherium simum simum	XP_004441608.1	XP_014636452.1	XP_004442687.1	XP_014645362.1	XP_004420216.1	XP_014649616.1
Cercocebus atys	XP_011942145.1	XP_011893527.1	XP_011949164.1	—	—	XP_011893112.1
Chaetura pelagica	—	XP_010004144.1	—	XP_009994230.1	KFU83910.1	XP_009997930.1
Charadrius vociferus	—	XP_009880208.1	—	—	XP_009889406.1	—
Chelonia mydas	—	XP_007062699.1	—	XP_007067100.1	XP_007053651.1	XP_007057964.1
Chinchilla lanigera	XP_005414100.1	XP_005379244.1	XP_013360585.1	XP_013368945.1	XP_005376559.1	XP_005375289.1
Chlorocebus sabaeus	—	XP_007973871.1	—	—	XP_008012737.1	—
Chrysemys picta bellii	—	XP_005302503.1	XP_005279608.1	XP_005307496.1	XP_005295945.1	XP_005311075.1
Chrysochloris asiatica	XP_006871474.1	XP_006865277.1	XP_006875157.1	XP_006864603.1	XP_006866164.1	XP_006834185.1
Ciona intestinalis	—	—	—	—	XP_009862126.1	—
Clupea harengus	—	—	XP_012694314.1	XP_012686788.1	XP_012689714.1	—
Colius striatus	—	—	—	—	XP_010202177.1	—
Colobus angolensis palliatus	XP_011807875.1	—	XP_011810238.1	—	—	—
Columba livia	—	XP_021138056.1	—	XP_005502561.1	XP_005503512.2	—
Condylura cristata	XP_004694187.1	XP_017593373.1	XP_004688795.1	XP_012580367.1	XP_004686812.1	XP_004685574.1
Corvus brachyrhynchos	—	—	—	XP_008636811.1	KFO58285.1	XP_008636237.1
Corvus cornix cornix	—	XP_019148111.1	—	—	—	—
Coturnix japonica	—	XP_015717966.1	—	XP_015716366.1	XP_015731168.1	XP_015740339.1
Cricetulus griseus	—	XP_003514245.1	—	—	XP_007614336.1	XP_003498896.1
Crassostrea virginica	—	—	—	XP_022318258.1	—	—
Cricetulus griseus	—	—	XP_003499782.1	XP_007638805.1	—	—
Crocodylus porosus	—	XP_019400405.1	—	XP_019390124.1	XP_019390681.1	—
Cuculus canorus	—	XP_009566732.1	—	XP_009563451.1	—	XP_009558874.1
Cyanistes caeruleus	—	XP_023791561.1	—	—	—	—
Cynoglossus semilaevis	XP_008307749.1	—	XP_008315818.2	XP_008315684.1	XP_016894992.1	XP_008317759.1
Cyprinodon variegatus	—	—	XP_015254374.1	XP_015239330.1	XP_015228777.1	—
Cyprinus carpio	—	—	—	—	—	XP_018964306.1
Danio rerio	XP_001920935.4	—	NP_001106948.1	XP_021329567.1	—	NP_001007329.1
Dasypus novemcinctus	—	—	—	—	XP_004450558.1	—
Delphinapterus leucas	XP_022441330.1	XP_022452419.1	—	—	—	XP_022417643.1
Desmodus rotundus	—	XP_024434983.1	—	—	—	—
Dipodomys ordii	XP_012888217.1	—	XP_012889414.1	XP_012881565.1	XP_012871485.1	—
Echinops telfairi	XP_004710557.1	XP_012859580.1	XP_004716860.1	XP_004717436.1	—	—
Egretta garzetta	—	XP_009646023.1	—	XP_009634645.1	—	—

Species	SHKBP1 (n = 124)	KCTD17 (n = 139)	KEAP1 (n = 135)	KLHL2 (n = 162)	KLHL3 (n = 158)	KLHL12 (n = 129)
Elephantulus edwardii	XP_006900961.1	XP_006890212.1	XP_006898831.1	XP_006896716.1	XP_006899557.1	—
Enhydra lutris kenyoni	XP_022380965.1	—	XP_022382690.1	XP_022370359.1	XP_022348825.1	XP_022364617.1
Eptesicus fuscus	XP_008137806.1	XP_008142891.1	XP_008156282.1	XP_008142537.1	XP_008140142.1	—
Equus asinus	XP_014715764.1	XP_014706708.1	XP_014721012.1	—	XP_014683945.1	—
Equus caballus	XP_003362363.2	XP_023487576.1	XP_001492331.3	XP_005607800.1	XP_001504377.2	XP_001495985.1
Equus przewalskii	XP_008532834.1	—	—	—	XP_008512037.1	XP_008524709.1
Erinaceus europaeus	XP_007536779.1	XP_007519412.1	XP_007526023.1	—	XP_007517633.1	XP_007522166.1
Esox lucius	XP_010890266.1	—	XP_010871089.1	—	XP_019901531.1	XP_010888908.1
Exaiptasia pallida	—	—	—	XP_020894482.1	—	—
Falco cherrug	—	—	—	—	XP_005440701.2	XP_005447275.1
Falco peregrinus	—	—	—	XP_005230556.1	XP_005242799.2	—
Felis catus	XP_003997840.1	XP_023113233.1	XP_023101067.1	—	XP_019688114.1	XP_019677561.1
Ficedula albicollis	—	XP_005039846.1	—	—	XP_016157084.1	XP_005059410.1
Fukomys damarensis	XP_010612769.1	—	—	XP_010628165.1	XP_010612413.1	KFO24396.1
Fulmarus glacialis	—	XP_009572356.1	—	—	—	—
Fundulus heteroclitus	XP_021164550.1	—	XP_012705937.1	XP_012725245.1	XP_021168459.1	XP_012730968.1
Galeopterus variegatus	—	—	XP_008561317.1	—	XP_008573655.1	—
Gallus gallus	—	XP_004937809.2	—	NP_001264749.1	XP_015149440.1	XP_015154220.1
Gavia stellata	—	XP_009813946.1	—	—	KFV59300.1	—
Gavialis gangeticus	—	XP_019370816.1	—	XP_019377290.1	XP_019361778.1	—
Gekko japonicus	—	XP_015281963.1	—	—	—	—
Geospiza fortis	—	XP_005421877.1	—	XP_005415620.1	XP_005418758.1	XP_005426852.1
Gorilla gorilla gorilla	—	XP_018874316.1	—	—	—	—
Haliaeetus leucocephalus	—	XP_010583711.1	—	—	XP_010577185.1	—
Haliaeetus albicilla	—	—	—	XP_009928870.1	—	—
Haplochromis burtoni	—	—	XP_014196664.1	—	—	—
Heterocephalus glaber	XP_004874267.1	XP_012927957.1	EHB12931.1	XP_004848282.1	XP_004836251.1	EHB03738.1
Hippocampus comes	—	—	XP_019717662.1	XP_019717542.1	XP_019739597.1	XP_019744013.1
Hipposideros armiger	XP_019485272.1	XP_019520201.1	XP_019514927.1	—	XP_019517118.1	XP_019493677.1
Homo sapiens	XP_006723537.1	NP_001269613.1	XP_011526754.1	NP_009177.3	NP_059111.2	NP_067646.1
Ictalurus punctatus	XP_017322144.1	XP_017339989.1	XP_017337726.1	XP_017316882.1	XP_017329595.1	XP_017324276.1
Ictidomys tridecemlineatus	XP_005336496.1	XP_005322340.1	XP_005336185.1	XP_013215786.1	XP_005333265.1	XP_005330387.1
Jaculus jaculus	—	XP_012804799.1	XP_004667509.1	XP_004657220.1	XP_004665149.1	—
Kryptolebias marmoratus	—	—	XP_017285965.1	XP_017261728.1	XP_017273010.1	XP_017294510.1
Labrus bergylta	XP_020514016.1	—	XP_020503327.1	XP_020502841.1	—	XP_020493361.1
Larimichthys crocea	XP_019133021.1	—	XP_019129802.1	XP_010747705.1	XP_010741230.1	XP_019108919.1
Lates calcarifer	—	—	XP_018520553.1	XP_018546403.1	XP_018549229.1	XP_018542865.1
Latimeria chalumnae	—	—	XP_005994373.1	—	XP_005991519.1	XP_005988446.1
Lepidothrix coronata	—	—	—	XP_017661316.1	XP_017683358.1	XP_017676878.1
Lepisosteus oculatus	XP_006627705.1	—	—	—	—	XP_015197756.1
Leptonychotes weddellii	—	XP_006751188.1	XP_006741120.1	—	—	—
Leptosomus discolor	—	—	—	XP_009950311.1	KFQ10383.1	KFP98732.1

Species	SHKBP1 (n = 124)	KCTD17 (n = 139)	KEAP1 (n = 135)	KLHL2 (n = 162)	KLHL3 (n = 158)	KLHL12 (n = 129)
Limosa lapponica baueri	—	—	—	—	—	PKU41125.1
Lingula anatina	—	—	—	—	XP_013388754.1	—
Lipotes vexillifer	XP_007458702.1	XP_007454129.1	—	XP_007471591.1	XP_007461459.1	XP_007456221.1
Lonchura striata domestica	—	XP_021409774.1	—	XP_021405931.1	XP_021407958.1	—
Loxodonta africana	—	XP_023415713.1	XP_003413141.1	XP_010593899.1	XP_010589155.1	XP_003410281.1
Macaca fascicularis	XP_005589339.1	XP_005567444.1	—	—	—	—
Macaca mulatta	XP_014979475.1	NP_001252736.1	—	XP_001100501.1	NP_001252766.1	—
Macaca nemestrina	XP_011763031.1	XP_011710770.2	—	XP_011714137.1	XP_011714779.1	—
Manacus vitellinus	—	—	—	XP_008931371.1	KFW78569.1	XP_008927962.2
Mandrillus leucophaeus	XP_011839136.1	—	XP_011849866.1	XP_011823166.1	XP_011824557.1	—
Manis javanica	XP_017528133.1	XP_017508523.1	XP_017495620.1	XP_017511714.1	XP_017513581.1	XP_017514597.1
Marmota marmota marmota	XP_015352327.1	—	—	XP_015341732.1	—	—
Maylandia zebra	XP_004565061.1	—	XP_004567922.1	XP_004549335.1	XP_004553661.1	XP_004546484.1
Meleagris gallopavo	—	XP_019470588.1	—	XP_010707769.1	—	—
Melopsittacus undulatus	—	—	—	XP_005145559.1	—	—
Meriones unguiculatus	XP_021506303.1	XP_021492399.1	XP_021504873.1	XP_021493522.1	—	—
Merops nubicus	—	—	—	—	KFQ31548.1	—
Mesitornis unicolor	—	XP_010188342.1	—	—	—	—
Mesocricetus auratus	XP_005086624.1	XP_005067012.1	XP_005078609.1	XP_005075242.1	XP_021087667.1	—
Microcebus murinus	XP_012630007.1	XP_012642587.1	XP_012646096.1	XP_012626034.1	XP_020137732.1	XP_012599823.1
Microtus ochrogaster	XP_005371646.1	XP_005354301.1	XP_005346939.1	XP_005367805.1	—	XP_005348437.1
Miniopterus natalensis	XP_016064707.1	—	XP_016063423.1	XP_016053241.1	XP_016059235.1	—
Mizuhopecten yessoensis	—	—	—	XP_021358880.1	—	—
Monodelphis domestica	XP_007492042.1	XP_016281346.1	XP_007488618.1	XP_007496215.1	XP_007473357.1	—
Monopterus albus	—	—	XP_020452724.1	XP_020476449.1	XP_020463137.1	XP_020473490.1
Mus caroli	XP_021021679.1	XP_021038727.1	XP_021027535.1	—	—	—
Mus musculus	XP_006539730.2	XP_006521523.2	NP_057888.1	XP_017168500.1	XP_006517071.1	NP_001298065.1
Mus pahari	XP_021049670.1	XP_021072585.1	XP_021062655.1	—	XP_021071267.1	XP_021053254.1
Mustela putorius furo	XP_004776173.1	XP_012907038.1	XP_004748492.1	XP_004779602.2	XP_012907923.1	XP_012913608.1
Myotis brandtii	XP_005868856.1	—	XP_005859245.1	XP_014390466.1	XP_005881064.1	XP_005886426.1
Myotis davidii	XP_006759262.1	—	ELK28701.1	XP_015424193.1	—	XP_006757531.1
Myotis lucifugus	—	XP_006107324.2	XP_006104108.1	XP_014311670.1	—	—
Nannospalax galili	XP_008852225.1	XP_008849546.1	XP_008848262.1	XP_008829715.1	XP_008830034.1	XP_008828554.1
Nanorana parkeri	—	—	XP_018431113.1	—	—	XP_018412396.1
Neolamprologus brichardi	XP_006801280.1	—	XP_006787448.1	—	—	XP_006794161.1
Neomonachus schauinslandi	XP_021556625.1	—	—	—	—	—
Neophocaena asiaeorientalis asiaeorientalis	XP_024624432.1	XP_024617351.1	—	—	—	—
Nestor notabilis	—	XP_010017253.1	—	—	—	—
Nipponia nippon	—	XP_009473450.1	—	XP_009473047.1	KFQ93934.1	—
Nomascus leucogenys	XP_012352070.1	XP_003264762.1	—	XP_003257978.1	XP_003266467.1	XP_003264617.1
Nothobranchius furzeri	XP_015806312.1	—	—	XP_015797306.1	—	XP_015824029.1
Notothenia coriiceps	—	—	XP_010766987.1	XP_010789689.1	—	—

Species	SHKBP1 (n = 124)	KCTD17 (n = 139)	KEAP1 (n = 135)	KLHL2 (n = 162)	KLHL3 (n = 158)	KLHL12 (n = 129)
Numida meleagris	—	XP_021241564.1	—	XP_021250584.1	—	XP_021232836.1
Ochotona princeps	XP_004598066.1	XP_004589821.2	XP_004600089.2	XP_004591480.1	XP_004586491.1	XP_004578811.1
Octodon degus	XP_004648039.1	—	XP_004632901.1	XP_004630664.1	XP_004632291.1	XP_004625419.1
Octopus bimaculoides	—	—	—	XP_014770021.1	—	—
Odobenus rosmarus divergens	XP_004396404.1	—	XP_004398925.1	XP_004411320.2	XP_004397909.1	XP_004392834.2
Odocoileus virginianus texanus	XP_020759499.1	—	XP_020758035.1	—	XP_020748682.1	XP_020759424.1
Oncorhynchus kisutch	XP_020363280.1	—	—	—	XP_020357787.1	XP_020315264.1
Oncorhynchus mykiss	XP_021443142.1	—	XP_021412413.1	—	XP_021439271.1	XP_021421846.1
Oncorhynchus tshawytscha	XP_024300321.1	—	—	—	—	—
Ophiophagus hannah	—	—	ETE73917.1	—	—	ETE69140.1
Orbicella faveolata	—	—	—	XP_020601220.1	—	—
Orcinus orca	XP_004271317.1	XP_012392053.1	XP_004277407.1	XP_004284035.1	XP_004282135.1	XP_012394372.1
Oreochromis niloticus	XP_013129084.1	—	XP_003447974.1	XP_019215626.1	XP_003443442.1	XP_005448525.1
Orycteropus afer afer	XP_007941168.1	XP_007939819.1	XP_007951766.1	XP_007952393.1	XP_007937122.1	XP_007939336.1
Oryctolagus cuniculus	—	—	XP_002723322.1	XP_008271730.1	XP_008253281.1	XP_002717633.2
Oryzias latipes	XP_023817440.1	—	XP_020557948.1	XP_020559732.1	XP_004073059.3	XP_004070913.1
Oryzias melastigma	XP_024133077.1	—	—	—	—	—
Otolemur garnettii	XP_003802151.1	XP_003783288.1	XP_003798009.1	—	XP_003782213.1	—
Ovis aries	—	—	—	XP_011952679.1	XP_004008868.1	XP_012043275.1
Ovis aries musimon	XP_011986482.1	XP_012004229.1	XP_011991418.1	—	XP_011971358.1	—
Pan paniscus	XP_003812529.1	XP_003821599.2	XP_003809635.1	—	XP_003829282.1	XP_014198142.1
Pan troglodytes	XP_016791502.1	XP_001160085.2	NP_001266890.1	XP_001150117.3	XP_016810041.1	—
Panthera pardus	XP_019287597.1	XP_019302361.1	—	—	XP_019279815.1	—
Panthera tigris altaica	—		XP_007079390.1	—	XP_007077902.1	—
Pantholops hodgsonii	XP_005963560.1	XP_005957223.1	—	—	—	—
Papio anubis	XP_017807946.1	XP_003905544.2	XP_003914932.1	—	—	—
Paralichthys olivaceus	—	—	XP_019939843.1	XP_019956734.1	XP_019941392.1	XP_019960243.1
Paramormyrops kingsleyae	XP_023668026.1	—	—	—	—	—
Parus major	—	XP_015483734.1	—	XP_015480275.1	—	XP_015506363.1
Patagioenas fasciata monilis	—	—	—	—	OPJ88162.1	OPJ82394.1
Pelecanus crispus	—	XP_009486864.1	—	—	—	—
Pelodiscus sinensis	—	XP_014434404.1	—	—	—	XP_006135792.1
Peromyscus maniculatus bairdii	XP_006995894.1	XP_006979168.2	XP_006987077.1	XP_015861554.1	XP_006976813.2	XP_006982523.1
Phascolarctos cinereus	—	XP_020838182.1	XP_020850692.1	—	—	—
Phalacrocorax carbo	—	—	—	KFW84676.1	—	—
Phascolarctos cinereus	—	—	—	XP_020831993.1	—	—
Phoenicopterus ruber ruber	—	KFQ81133.1	—	—	—	—
Physeter catodon	XP_007128153.1	XP_007111830.1	XP_007107317.1	—	XP_007112795.1	XP_007121223.1
Picoides pubescens	—	XP_009900889.1	—	XP_009902865.1	KFV61276.1	—
Piliocolobus tephrosceles	XP_023084903.1	XP_023077918.1	—	—	—	—
Pleuragramma antarctica	—	—	SCW25703.1	—	—	—
Poecilia formosa	XP_007570740.1	—	XP_007551655.1	XP_007549688.1	XP_007577950.1	XP_007553643.1

Species	SHKBP1 (n = 124)	KCTD17 (n = 139)	KEAP1 (n = 135)	KLHL2 (n = 162)	KLHL3 (n = 158)	KLHL12 (n = 129)
Poecilia latipinna	XP_014915223.1	—	XP_014907996.1	XP_014915653.1	XP_014902577.1	—
Poecilia mexicana	—	—	XP_014837107.1	XP_014829877.1	XP_014860420.1	XP_014840751.1
Poecilia reticulata	XP_008400256.1	—	XP_008413531.1	XP_008400816.1	—	XP_008412643.1
Pogona vitticeps	—	XP_020643409.1	XP_020654350.1	XP_020657605.1	XP_020638337.1	XP_020638148.1
Pongo abelii	XP_024092291.1	XP_003779591.1	NP_001126406.1	XP_002815314.1	NP_001127192.1	—
Propithecus coquereli	XP_012512503.1	XP_012493505.1	XP_012496759.1	—	XP_012501789.1	—
Protobothrops mucrosquamatus	—	XP_015687420.1	—	XP_015676122.1	—	XP_015671067.1
Pseudopodoces humilis	—	XP_005527632.2	—	XP_005517662.1	XP_005523584.1	XP_005530239.1
Pterocles gutturalis	—	—	—	XP_010076229.1	—	—
Pteropus alecto	XP_006905479.1	XP_006914536.1	—	—	—	XP_006908363.1
Pteropus vampyrus	XP_011370513.1	XP_011385740.1	XP_011378571.1	XP_011362014.1	XP_011360672.1	—
Pundamilia nyererei	—	—	XP_005752286.1	—	XP_005726524.1	XP_005731643.1
Pygocentrus nattereri	XP_017537184.1	—	XP_017548442.1	XP_017572936.1	XP_017545599.1	XP_017548954.1
Python bivittatus	—	XP_007421076.1	XP_007426630.1	—	—	XP_007426111.1
Rattus norvegicus	NP_001258009.1	XP_008763932.2	NP_476493.2	XP_008770267.1	—	XP_006249905.1
Rhinolophus sinicus	XP_019606931.1	XP_019575200.1	XP_019600540.1	XP_019600193.1	XP_019595782.1	XP_019606448.1
Rhinopithecus roxellana	XP_010379143.1	XP_010387491.1	XP_010372068.1	XP_010362880.1	XP_010386363.1	—
Rousettus aegyptiacus	—	XP_016017310.1	XP_016009006.1	XP_016010073.1	XP_016016102.1	—
Saccoglossus kowalevskii	—	—	—	XP_002730487.1	—	—
Saimiri boliviensis boliviensis	XP_010329404.1	XP_003932948.2	XP_010347939.1	XP_010330383.1	XP_003934008.1	XP_010347190.1
Salmo salar	XP_014017560.1	XP_014034374.1	XP_013986100.1	—	XP_014068166.1	XP_014002172.1
Salvelinus alpinus	XP_023838330.1	—	—	—	—	—
Sarcophilus harrisii	—	—	XP_003760514.1	—	—	XP_003767642.1
Scleropages formosus	XP_018598136.1	—	KPP71934.1	XP_018591491.1	XP_018589121.1	XP_018597687.1
Serinus canaria	—	—	—	—	XP_009089645.1	XP_009095577.1
Seriola dumerili	XP_022594116.1	—	XP_022622580.1	XP_022593877.1	XP_022615920.1	XP_022618872.1
Seriola lalandi dorsalis	XP_023256419.1	—	XP_023274978.1	XP_023265271.1	XP_023253862.1	—
Sinocyclocheilus anshuiensis	—	—	XP_016349766.1	XP_016299079.1	XP_016310893.1	XP_016330970.1
Sinocyclocheilus grahami	—	—	XP_016109850.1	XP_016136233.1	XP_016145128.1	XP_016094876.1
Sinocyclocheilus rhinocerous	XP_016369534.1	—	XP_016364948.1	XP_016414377.1	XP_016409147.1	XP_016372389.1
Sorex araneus	XP_004620791.1	XP_004610551.2	XP_004616619.1	XP_004610419.1	XP_012788506.1	XP_004610091.1
Stegastes partitus	—	—	XP_008275180.1	XP_008291203.1	XP_008303524.1	XP_008275958.1
Strongylocentrotus purpuratus	—	—	—	—	XP_011677555.1	—
Struthio camelus australis	—	XP_009682597.1	—	XP_009675470.1	—	—
Sturnus vulgaris	—	—	—	—	XP_014738920.1	—
Stylophora pistillata	—	—	—	XP_022807913.1	—	—
Sus scrofa	XP_020950056.1	XP_020947187.1	NP_001108143.1	XP_005666707.1	XP_020940448.1	XP_003130659.3
Taeniopygia guttata	—	XP_012424696.1	—	XP_002197028.2	XP_002187183.3	—
Takifugu rubripes	XP_011615817.1	—	XP_003972643.1	XP_003972526.2	XP_011609237.1	XP_003963586.1
Tauraco erythrolophus	—	—	—	KFV09010.1	—	—
Thamnophis sirtalis	—	—	—	—	XP_013925240.1	—
Tinamus guttatus	—	XP_010222429.1	—	—	—	—

Species	SHKBP1 (n = 124)	KCTD17 (n = 139)	KEAP1 (n = 135)	KLHL2 (n = 162)	KLHL3 (n = 158)	KLHL12 (n = 129)
Trichechus manatus latirostris	XP_004390909.1	XP_004373880.1	XP_004378408.1	XP_004390697.1	XP_004384746.1	XP_004375335.1
Tupaia chinensis	XP_006140529.1	XP_014446395.1	XP_006161600.1	—	—	XP_006159222.1
Tursiops truncatus	—	XP_019774047.1	—	—	—	—
Tyto alba	—	—	—	—	KFV43663.1	—
Ursus maritimus	—	—	—	XP_008683826.1	—	—
Vicugna pacos	XP_006215068.1	XP_015097025.1	XP_006206340.1	XP_006213249.1	XP_006204669.1	XP_006213306.2
Xenopus laevis	XP_018084584.1	—	XP_018111773.1	XP_018097193.1	—	XP_018104893.1
Xenopus tropicalis	XP_002938848.1	—	NP_001008024.1	XP_002934798.2	—	—
Xiphophorus maculatus	XP_014327689.1	—	XP_023189591.1	XP_005809438.1	XP_023184792.1	XP_023187727.1
Zonotrichia albicollis	—	XP_014123680.1	—	XP_005486355.1	—	—

The list of species and GenBank entries contained only protein sequences: *i*) having an unambiguous description (*i.e.* including the name of the queried KCTD or BTB-Kelch protein); *ii*) that aligned with ≥ 80% sequence coverage and had ≥ 60% sequence identity with the query sequence; *iii*) that contained all the annotated domains (using the domain annotation automatically generated by the Uniprot Knowledgebase); and *iv*) without any gap in positions of the conserved domains. Proteins that did not pass these filtering steps were discarded.
