# Supplementary material for: Comparative structural and evolutionary analyses predict functional sites in the artemisinin resistance malaria protein K13
Source: Sci Rep. 2019 Jul 23;9:10675. doi: 10.1038/s41598-019-47034-6 (PMC6650413; doi:10.1038/s41598-019-47034-6)
Supplement: Supplementary file 3 — Supplementary Methods [file 41598_2019_47034_MOESM3_ESM.pdf]

Supplementary Methods

This supplemental file has been provided by the authors to give readers additional information about their work.

Supplement to: **Comparative structural and evolutionary analyses predict functional sites in the artemisinin resistance malaria protein K13**

By Romain Coppée, Daniel C. Jeffares, Maria A. Miteva, Audrey Sabbagh, Jérôme Clain

Method S1 .... **2**

Method S2 .... **3**

Method S3 .... **3**

References **5**

Materials and Methods S1. Description of PAML models and interpretations

The heterogeneity of ω among lineages of the *k13* phylogenetic tree (branch models) was tested by comparing the free-ratio (FR) model, which assumes as many ω parameters as the number of branches in the tree, to the one-ratio (M0) model which supposes only one ω value for all branches.[1,2]
M1a allows codon sites to fall into two site classes, either with $\omega < 1$ (purifying selection) or $\omega = 1$ (neutral evolution), whereas model M2a extends model M1a with a further site class as $\omega > 1$ (positive selection). Model M3 includes a discrete distribution of independent ω with k classes of sites (k = [3, 4, 5] in this study), with ω values and corresponding proportions estimated from the dataset. Model M7 assumed a β-distribution of ten ω ratios limited to the interval [0, 1] with two shape parameters p and q, whereas model M8 adds an additional site class with ω possibly > 1 as M2a does. The heterogeneity of ω across codon sites was tested by comparison of models M0-M3, while comparison of paired models M1a-M2a and M7-M8 allowed to detect positive selection.[1]

Model comparisons were made using likelihood ratio tests (LRTs).[3] For each of the LRTs, twice the log-likelihood difference between alternative and null models ($2\Delta\ell$) was compared to critical values from a chi-squared distribution with degrees of freedom equal to the difference in the number of estimated parameters between both models.[4] Candidate sites for positive selection were pinpointed using the Bayes empirical Bayes (BEB) inference which calculates the posterior probability that each codon site falls into a site class affected by positive selection (in models M2a and M8), as described by Yang and colleagues.[5] For model M3, in which no BEB approach is implemented yet, the Naïve empirical Bayes (NEB) approach was used to identify those sites evolving under positive selection.

Three codon substitution models were used and compared for all models: F1x4 and F3x4, which assume equal nucleotide frequencies and individual codon frequencies at all codon positions, respectively, and the parameter-rich model F61, which estimates codon frequencies separately for each codon.[6,7] Since the three codon substitution models yielded similar results ($p < 0.01$ in each pairwise comparison, Spearman's rank correlation), we only presented those obtained with the most widely used F3x4 codon model. The analyses were run multiple times with different ω starting values to check the consistency of the results.

For PAML model M3 with k site classes of ω ratios, the posterior mean of ω value at each codon site was calculated as the average of the ω ratios across the k ω site classes weighted by their posterior probabilities.[8]

Materials and Methods S2. Setting up of KCTD and BTB-Kelch datasets

Each *Homo sapiens* KCTD and BTB-Kelch protein sequence was successively submitted as query sequence for a blastp search[9] (BLOSUM62 scoring matrix, max target sequences fixed at 1,000) against the NCBI non-redundant protein database to retrieve orthologous sequences from a large amount of species. The output lists were then filtered according to specific criteria so as to keep only protein sequences having an unambiguous description (*i.e.* a description that includes the name of the queried KCTD or BTB-Kelch protein), and that aligned with ≥ 80% sequence coverage and had ≥ 60% sequence identity with the query sequence. The multiple protein sequence alignment of each set of orthologous sequences was then generated using MAFFT version 7[10] (E-INS-I strategy with BLOSUM62 scoring matrix, gap opening penalty 2.0 and offset 0.1). A second filtering step was performed to remove incomplete or miss-annotated sequences, *i.e.* the sequences that did not contain all the annotated domains (using the domain annotation automatically generated by the Uniprot Knowledgebase) and/or that included a gapped position located in one of the annotated domains. The final multiple protein sequence alignments included: *i*) 124 sequences × 103 aligned positions for SHKBP1; *ii*) 139 sequences × 102 aligned positions for KCTD17; *iii*) 135 sequences × 285 aligned positions for KEAP1; *iv*) 162 sequences × 286 aligned positions for KLHL2; *v*) 158 sequences × 286 aligned positions for KLHL3; and *vi*) 129 sequences × 289 aligned positions for KLHL12. The full list of orthologous sequences used for each mammalian KCTD and BTB-Kelch protein is provided in Table S6.

Materials and Methods S3. Molecular dynamics simulations set up

At startup, the K13 KREP crystallographic structure was retrieved from the PDB repository, PDB ID: 4ZGC.[11] One monomer (chain B) and the BTB domain of the two monomers were excluded for molecular dynamics simulations. In order to evaluate the influence of ART-R mutations on the PfK13

KREP fold, three models were generated: (*i*) K13 KREP harboring the most common C580Y mutation in natural populations; (*ii*) K13 KREP harboring the R539T mutation which confers the highest level of ART-R; and (*iii*) K13 KREP harboring the A578S mutation which serves as control since the mutation do no confer ART-R. Single mutations were introduced using the *swapaa* function of UCSF Chimera by substituting the residue with the most probable rotameric conformation.[12] All missing atoms were then added using Swiss PDB Viewer.[13] Generated models were checked for quality using MolProbity[14] which showed no outliers and > 98% of residues (including *in silico* introduced mutations) in favored regions. Molecular dynamics simulations were carried out using the GROMACS package, v. 5.0.7.[15] with the improved side-chain torsion potentials force field Amber99ss-ILDN for amino acid interaction.[16] Protein systems were immersed in a dodecahedron box of TIP3P water molecules[17] preserving at least 13 Å of separation between the solute and the edges of the box. The Particle Mesh Ewald (PME)[18] approach was employed with van der Waals and Coulomb non-bonded interactions truncated at 10 Å. Bond lengths were constrained using the LINCS algorithm[19] that allowed a 2 fs time step in all simulations. The ionization state of residues was set to be consistent with neutral pH, and counter-ions Na^+ were then added by randomly replacing water molecules to ensure the overall charge neutrality of the system. The whole systems consisted of ~35 000 atoms. To release conflicting contacts, solvated systems were subjected to energy minimization using the steepest descent algorithm over 5 000 steps until the maximum force < 1000.0 kJ/mol^{-1}/nm^{-1}. Before MD productions, each solvated system was subjected to two-step equilibration. In the first step, systems were equilibrated for 100 ps in the NVT ensemble at 300 K with the V-rescale temperature coupling.[20] The equilibrated systems from the NVT ensemble were then treated to constant pressure (NPT) ensemble for 100 ps using the Parrinello-Rahman barostat[21] under an isothermal-isobaric pressure of 1.0 bar. Position restrains were applied to all atoms during equilibration steps to avoid configuration changes. MD productions were run for 100 ns in the absence of any restraints. During MD productions, the V-rescale thermostat coupled with the Parrinello-Rahman barostat were used to maintain the temperature and pressure at 300 K and 1.0 bar, respectively. The trajectories were stored at every 10 ps. Each wild type and mutant system was subjected to three molecular dynamics replicates with the same parameters and algorithms but different velocities. MD trajectories were then

analyzed using inbuilt GROMACS tools. The first 5 ns of trajectories (which referred as equilibration time), were removed *prior* to analyses.

References:

1. Nielsen R, Yang Z. (1998) Likelihood models for detecting positively selected amino acid sites and applications to the HIV-1 envelope gene. Genetics 148: 929–936.

2. Yang Z. (1998) Likelihood ratio tests for detecting positive selection and application to primate lysozyme evolution. Mol Biol Evol 15: 568–573.

3. Vuong QH. (1989) Likelihood ratio tests for model selection and non-nested hypotheses. Econometrica 57: 307–333.

4. Yang Z, Nielsen R, Goldman N, Pedersen AM. (2000) Codon-substitution models for heterogeneous selection pressure at amino acid sites. Genetics 155: 431–449.

5. Yang Z, Wong WSW, Nielsen R. (2005) Bayes empirical bayes inference of amino acid sites under positive selection. Mol Biol Evol 22: 1107–1118.

6. Goldman N, Yang Z. (1994) A codon-based model of nucleotide substitution for protein-coding DNA sequences. Mol Biol Evol 11: 725–736.

7. Muse SV, Gaut BS. (1994) A likelihood approach for comparing synonymous and nonsynonymous nucleotide substitution rates, with application to the chloroplast genome. Mol Biol Evol 11: 715–724.

8. Yang Z. PAML 4: phylogenetic analysis by maximum likelihood. (2007) Mol Biol Evol 24: 1586–1591.

9. Altschul SF, Gish W, Miller W, Myers EW, Lipman DJ. (1990) Basic local alignment search tool. J Mol Biol 215: 403–410.

10. Katoh K, Standley DM. (2013) MAFFT multiple sequence alignment software version 7: improvements in performance and usability. Mol Biol Evol 30: 772–780.

11. Jiang DQ, Tempel W, Loppnau P, Graslund S, He H, et al. (2015) Crystal structure analysis of Kelch protein (with disulfide bond) from *Plasmodium falciparum*. Protein Data Bank.

12. Pettersen EF, Goddard TD, Huang CC, Couch GS, Greenblatt DM, et al. (2004) UCSF Chimera—A visualization system for exploratory research and analysis. J Comput Chem 25: 1605–1612.

13. Guex N, Peitsch MC. (1997) SWISS-MODEL and the Swiss-PdbViewer: an environment for comparative protein modeling. Electrophoresis 18:2714–2723.

14. Chen VB, Arendall WB, Headd JJ, Keedy DA, Immormino RM, et al. (2010) MolProbity: all-atom structure validation for macromolecular crystallography. Acta Crystallogr D Biol Crystallogr 66: 12–21.

15. Abraham MJ, Murtola T, Schulz R, Páll S, Smith JC, et al. (2015) GROMACS: High performance molecular simulations through multi-level parallelism from laptops to supercomputers. SoftwareX 1–2:19–25.

16. Lindorff-Larsen K, Piana S, Palmo K, Maragakis P, Klepeis JL, et al. (2010) Improved side-chain torsion potentials for the Amber ff99SB protein force field. Proteins 78:1950–1958.

17. Jorgensen WL, Chandrasekhar J, Madura JD, Impey RW, Klein ML. (1983) Comparison of simple potential functions for simulating liquid water. J. Chem. Phys. 79:926–935.

18. Darden T, York D, Pedersen L. (1993) Particle mesh Ewald: An N·log(N) method for Ewald sums in large systems. J. Chem. Phys. 98:10089–10092.

19. Hess B, Bekker H, Berendsen HJC, Fraaije JGEM. (1997) LINCS: A linear constraint solver for molecular simulations. J. Comput. Chem. 18:1463–1472

20. Bussi G, Donadio D, Parrinello M. (2007) Canonical sampling through velocity rescaling. J. Chem. Phys. 126:014101.

21. Parrinello M, Rahman A. (1981) Polymorphic transitions in single crystals: A new molecular dynamics method. J. Appl. Phys.52:7182–7190.
